# Reconciling predicted and measured viscosity parameters in high concentration therapeutic antibody solutions

**DOI:** 10.1080/19420862.2024.2438172

**Published:** 2024-12-11

**Authors:** Georgina Bethany Armstrong, Aisling Roche, William Lewis, Zahra Rattray

**Affiliations:** a Drug Substance Development, GlaxoSmithKline, Stevenage, UK; b Strathclyde Institute of Pharmacy and Biomedical Sciences, University of Strathclyde, Glasgow, UK; c Large Molecule Discovery, GlaxoSmithKline, Stevenage, UK

**Keywords:** Antibody, formulation, physicochemical descriptors, prediction, viscosity

## Abstract

Monoclonal antibody (mAb) solution viscosity in ultra-high concentration formulations is a key developability consideration in mAb development risk mitigation strategies that has implications for downstream processing and patient safety. Predicting viscosity at therapeutically relevant concentrations remains critical, despite the need for large mAb quantities for viscosity measurement being prohibitive. Using a panel of IgG1s, we examined the suitability of viscosity prediction and fitting models at different mAb test concentration regimes. Our findings caution against extrapolation from low concentration measurements, as they lack predictive ability for ultra-high concentration regimes. For the first time, we demonstrate the importance of analyte concentration range selection, and the need for bespoke viscosity model development.

## Introduction

Monoclonal antibodies (mAbs) represent an important therapeutic class, with autoinjector-based dosing gaining popularity for patient and carer administration. Injection of mAbs subcutaneously requires high solution concentrations (>100 mg/mL) and low-dose volumes to achieve dose-relevant target therapeutic effects.^1^

High-concentration mAbs are subjected to substantial molecular crowding at ultra-high concentrations, and the conformational flexibility of mAbs leads to increased intermolecular interactions resulting in elevated opalescence, higher protein aggregation risk, phase separation, and elevated solution viscosity.^2^ Viscosity, a fluid’s resistance to flow or rate of deformation, is mechanistically characterized by examining electroviscous effects, mAb molecular size, and surface potential distributions, with these factors determining the likelihood of protein–protein interactions (PPIs).^2,3^

Developed from colloidal principles, the primary electroviscous effect describes the distortion of the electrical double-layer in the ultra-dilute regime with varying ionic strength. Changes in counterions and the hydration shell around mAbs affect their hydrodynamic volume and Brownian motion.^4,5^ With the growing demand for ultra-high concentration mAbs, secondary electroviscous effects reduce mAb solubility while increasing their solution viscosity.^2,6^ Increased crowding and decreased inter-‘particle’ distance increase the pair interaction potential.^7^ The interaction potential is quantified by the second virial coefficient (B_22_) or diffusion interaction parameter (k_D_) that are correlated with solution-phase viscosity.^8,9^ However, B_22_ and k_D_ do not fully capture anisotropic interactions due to surface potential variations.

Beyond pair-wise interactions, cluster formation from soluble mAb oligomerization drives elevated viscosity for high concentration mAb solutions. Small-angle X-ray scattering experiments and coarse-grained computational simulations have revealed mechanisms of mAb self-assembly and microstructure formation, directly correlating with increased viscosity.^10–12^

The complexity of interactions contributing to high viscosity, along with manufacturability and injectability risks, has driven the development of *in silico* sequence and structure-based models. Regression and clustering models^13–15^ have identified correlations between molecular descriptors derived from three-dimensional homology constructs and high concentration mAb viscosity. To mitigate the risks of overfitting of small, non-diverse datasets, machine learning classification tools are used to categorize mAb viscosity risks.^11,16–18^

Considering manufacturability, product quality, and injectability risks of highly viscous mAb formulations, a variety of mitigation strategies have been investigated to improve the likelihood of mAb translation to the clinic.^19^

Currently, there is a knowledge gap in selecting appropriate viscosity prediction models for high mAb solution viscosity, with no prior cross-comparisons in the ultra-high mAb concentration regime. This study comprehensively assesses viscosity fit and prediction models for nine anti-IL-8 mAbs (eight mutant variants and wild-type (WT)) previously manufactured, focusing on high- and ultra-high mAb concentration regimes. Using a combined computational and experimental approach, we compare the effectiveness of these models for triaging mAb developability. Our findings highlight the necessity of measuring viscosity at dose relevant ultra-high concentrations and reveal the limitations of predictive models, low concentration hydrodynamic properties and *in silico* molecular descriptors.

## Materials and methods

*In silico* structural modeling and generation of molecular descriptors was performed in Molecular Operating Environment (MOE) software, version 2020.0901 (Chemical Computing Group, Montreal, Canada).

### Homology constructs of anti-IL-8 Fv structures

Homology models^19^ for nine anti-IL-8 antibody variable fragment (Fv) regions, including eight single-point mutants were constructed based on the anti-IL-8 IgG1 Fab domain crystal structure (PDB: 505B). Using the *Antibody modeller* feature (version 2020.0901) in MOE with default refinement and forcefield settings, we created homology constructs and introduced single-point mutations *via* the *Residue Scan* feature. The same methodology was applied to construct homology models for four in-house mAbs.

### In silico *molecular descriptors*

We computed sequence and structure based physicochemical descriptors using the *Protein Properties* tool and *Descriptors* Feature in *BioMOE* (version 2021-11-18, Chemical Computing Group, Montreal, Canada). Viscosity-relevant parameters for input into predictive models are reported in Supplementary Table S1.

### Aggregation propensity tools: TANGO and WALTZ.

The TANGO^20,21^ (http://tango.crg.es/tango.jsp.) and WALTZ^22,23^ (https://waltz.switchlab.org/) sequence-based aggregation propensity tools were used to predict cross-beta-sheet formation in all anti-IL-8 IgGs examined.

### DeepSCM

A convolutional neural network (https://github.com/Lailabcode/DeepSCM.) was used to assess charge distributions of the assumed Fv structure over molecular dynamic simulations.^17,24^ All anti-IL-8 heavy and light chain variable sequences were inputted separately as FASTA files and the code was run in the terminal on a Linux system.

### Viscosity prediction from Fv construct molecular descriptors

Three empirical models derived from the regression of viscosity data and molecular descriptors were used to directly predict viscosity at either 150 mg/mL^13^ or 180 mg/mL.^9,14^

The *viscosity model* by Li *et al*. uses the structure-based isoelectric point and WALTZ aggregation propensity score, normalized by the number of amino acid residues to generate relative viscosity predictions.^13^

(1) $$\frac{\ln\left(\eta_{\mathrm{rel}}\right)}{N_{\mathrm{re}s_{\mathrm{Fv}}}}=0.022182-0.55131\times \left(\frac{pI_{3D}}{N_{\mathrm{re}s_{\mathrm{Fv}}}}\right)+0.00087416\times \left(\frac{P_{\mathrm{aggWALTZFv}}}{N_{\mathrm{re}s_{\mathrm{Fv}}}}\right)$$

where $\eta_{\mathrm{rel}}$ is the relative viscosity, $N_{\mathrm{re}s_{\mathrm{Fv}}}$ the number of residues in the Fv (*N* = 227, anti-IL-8 mutant panel), $pI_{3D}$ the structure-based isoelectric point, computed from homology constructs in MOE, and $P_{\mathrm{aggWALTZFv}}$ the WALTZ aggregation propensity score for the Fv construct.

The *Sharma viscosity model* incorporated a hydrophobic index score (*HI*) and the Fv charge symmetry (*F*_*vCSP*_) to account for nonpolar attractive interactions, and repulsive interactions arising from net charge.^14^

(2A) $$\eta =10^{\left(\left(0.15+1.26\left(0.6\right)\right)HI-0.043F_{v}\mathrm{charge}-0.02\left(0.015\right)F_{\mathrm{vCSP}}\right)}$$

(2B) $$\mathrm{HI}=-\left(\frac{\Sigma n_{i}E_{i}}{\Sigma n_{j}E_{j}}\right)$$

where η is the dynamic viscosity, FvCSP is the Fv charge symmetry, and HI the hydrophobic index, calculated using Equation 3B. n_i_ represents the number of hydrophobic amino acids (i.e., A, C, F, I, L, P, V, G, W, and Y), and n_j_ the hydrophilic amino acids (i.e., D, E, H, K, M, N, Q, R, S, and T). E is the Eisenberg hydrophobicity score for each residue.^25^

Tomar et al. developed an empirical viscosity prediction model from the regression of molecular descriptors for 16 mAbs.^15^

(3A) $$\ln\frac{\eta }{\eta_{0}}=-0.58+\mathrm{Bc}$$

(3B) $$B=-0.0044\times pI_{3D}+0.056$$

where η is the dynamic viscosity and $\eta_{0}$ the buffer viscosity. −0.58 is used as the average value of intercept of the slope (B) when $\ln\frac{\eta }{\eta_{0}}$ is plotted against antibody concentration (c), which is 180 mg/mL in the original study.^15^

The *Tomar* model Equation 3A was used to fit concentration–viscosity profiles, using parameterization with experimental viscosity measurements to interpolate/extrapolate viscosity at different concentrations (Table 1).

**Table 1.** Viscosity model equations. η is the dynamic viscosity (cP), and c the concentration (mg/mL). For the exponential growth equation, Y_0_ the intercept (cP), k the rate constant (mL/mg). For the three-parameter exponential equation, α_2_ the slope for concentration versus ln(η) and α_3_ the slope for 1 divided by the temperature (K^−1^) versus $\ln\left(\eta \right)$, and α_1_ the intercept (cP). For the modified Ross-Minton model, $\eta_{0}$ represents the buffer viscosity (cP) set at 1.13, $\left[\eta \right]$ the intrinsic viscosity, *k* the crowding factor, *v* the simha shape parameter. $\left[\eta \right]$, *k* and *v* were estimated using the Generalized Reduced Gradient non-linear solver function to find the local optimum value to reduce the sum of squared errors. Finally, for the tomar model $\ln\left(A\right)$ is the intercept of the slope B, when $\ln\frac{\eta }{\eta_{0}}$ is plotted against concentration. To find the knee of each exponential model, each equation was solved with the first derivative ($\frac{\mathrm{d\eta}}{\mathrm{dc}}$) set to 1. For the Ross Minton model, this required solving for *c* numerically, using the generalized reduced gradient non-linear solver function with the objective of $\frac{\mathrm{d\eta}}{\mathrm{dc}}$ = 1.

Model name	*Exponential growth*	*Three-parameter exponential*	*Modified Ross-Minton*^2^	*Tomar*^9,15^
Equation	$\eta =Y_{0}e^{\mathrm{kC}}$	$\eta =e^{\left(a_{1}+a_{2}*c+\frac{a_{3}}{T}\right)}$	$\eta =\eta_{0}\exp\left(\frac{\left[\eta \right]c}{1-\left(\frac{\kappa }{\upsilon }\right)\left[\eta \right]c}\right)$	$\ln\frac{\eta }{\eta_{0}}=\ln A+\mathrm{Bc}$
References	Simple formula to describe concentration-dependent viscosity^26,27^ The exponential coefficient, *k*, can be used to simplify correlations to other hydrodynamic/biophysical parameters. Used for viscosity prediction in neural networks^16^	In-house model developed from simplification of an empirical model^28^	A widely used formula, derived from Mooney’s semi-empirical equation relating effective volume fraction to intrinsic viscosity.^29^ Used in fitting viscosity curves^30,31^ as well as deriving viscosity and hydrodynamic parameters.^4,31,32^	Linearized exponential derived from prediction of viscosity curves on a dataset of 16 mAbs.^15^
Knee of curve equation	$c=\frac{\ln\frac{1}{\Upsilon_{0}k}}{k}$	$c=\frac{\left(\ln\left(\frac{1}{a_{2}}\right)\right)-a_{1}-a_{3}}{a_{2}}$	$\frac{\mathrm{d\eta}}{\mathrm{dc}}=1=\eta \times \left(\frac{\left[\eta \right]}{1-\left(\frac{\kappa }{\nu }\right)\times \eta \times c}\right)$	$c=\frac{\left(\ln\frac{1}{B\eta_{0}A}\right)}{B}$

### Protein expression and purification

An anti-IL-8 IgG panel was generated in a Chinese Hamster Ovary glutamine-synthetase-knockout cell line.^19^ Cation exchange polishing followed before diafiltration by small-scale tangential flow filtration (pH monitored and remained at pH 6.0 (±0.2) throughout concentration and diafiltration steps) and concentration to ≥150 mg/mL in a proprietary histidine-based formulation buffer containing trehalose and arginine (pH 6.0, 0.1 M ionic strength). Identity and purity was confirmed for each molecule.^19^ All samples for low concentration analytics were prepared from the dilution of the highly concentrated mAb product.

### Viscosity measurement

A VROC Initium (Rheosense, United States) was used to measure viscosity using the ‘Auto’ shear rate function at fixed shear rates (100, 180, 500, 1000, and 2000s^−1^). Newtonian behavior was observed across all IgGs, with consistent viscosities recorded across shear rates tested (averages reported). Sample viscosity was measured up to 260 mg/mL to derive viscosity–concentration profiles. Viscosity data were segmented into two concentration regimes: high (≤120 mg/mL)^19^ and ultra-high (≤260 mg/mL, inclusive of high concentration data). Data were subjected to the following criteria: priming segments were excluded, the percent full scale was in the 5–95% range, the R^2^ fit of the pressure sensor position was ≥0.998, and transient curves reached steady plateaus with no drift.

### Viscosity parameter fitting

An exponential growth, a simplified three-parameter exponential, a modified Ross-Minton and Tomar viscosity models were used to fit viscosity–concentration data for the mAb panel to determine the optimal model (Table 1).

#### Intrinsic viscosity calculation

For intrinsic viscosity $\left[\eta \right]$ measurements multiple priming segments were set up, followed by 10 replicates at the maximum shear rate (23,080 s^−1^). Formulation buffer and test mAbs (5–50 mg/mL) were measured to determine the relative viscosities ($\eta_{\mathrm{rel}}$) (Equation 4), from which the specific ($\eta_{\mathrm{sp}}$) (Equation 5) and reduced viscosities ($\eta_{\mathrm{red}}$) (Equation 6) could be calculated.

(4) $$\eta_{\mathrm{rel}}=\frac{\eta }{\eta_{0}}$$

where the relative viscosity ($\eta_{\mathrm{rel}}$), (cP), is the apparent dynamic viscosity of the sample η (cP), divided by the apparent dynamic viscosity of the formulation buffer only, $\eta_{0}$ (cP).

(5) $$\eta_{\mathrm{sp}}=\eta_{\mathrm{rel}}-1$$

where the specific viscosity$(\eta_{\mathrm{sp}}$) (cP), which can be used to calculate the reduced viscosity ($\eta_{\mathrm{red}})$, (cP):

(6) $$\eta_{\mathrm{red}}=\frac{\eta_{\mathrm{sp}}}{c}$$

where *c* is the mAb solution concentration (mg/mL).

The Huggins equation describes the concentration-dependence of $\eta_{\mathrm{red}}$ in the dilute concentration linear region:

(7) $$\eta_{\mathrm{red}}=\left[\eta \right]+k_{H}{\left[\eta \right]}^{2}c$$

where [η] is the intrinsic viscosity (mL/g), *k*_*H*_ is the Huggins coefficient.

Slopes and intercepts from the linear regression of $\eta_{\mathrm{red}}$ versus the mAb concentration range were used to compute the Huggins coefficient (*k*_*H*_):

(8) $$k_{H}=\frac{\mathrm{slopef}\left(c\right)}{{{\left[\eta \right]}_{\mathrm{avg}}}^{2}}$$

The intrinsic viscosity ${\left[\eta \right]}_{\mathrm{avg}}$ was determined from the intercept.

The uncertainty of *k*_*H*_ was calculated from the propagation of the error equation:

(9) $$k_{H}\times \sqrt{{\left(\frac{\mathrm{\sigma x}}{x}\right)}^{2}+{\left(\frac{\left(\sigma {{\left[\eta \right]}_{\mathrm{avg}}}^{2}\right)}{\left({{\left[\eta \right]}_{\mathrm{avg}}}^{2}\right)}\right)}^{2}-2\frac{\left(\sigma {{\left[\eta \right]}_{\mathrm{avg}}}^{2}\right)\times \left(\mathrm{\sigma x}\right)}{({{\left[\eta \right]}_{\mathrm{avg}}}^{2})\times x}}$$

where $k_{H}$ is the Huggins coefficient, ${{\left[\eta \right]}_{\mathrm{avg}}}^{2}$ the squared intrinsic viscosity from either respective linear regression, $\sigma {{\left[\eta \right]}_{\mathrm{avg}}}^{2}$ the error of squared intrinsic viscosity, calculated as $\sigma {{\left[\eta \right]}_{\mathrm{avg}}}^{2}={{\left[\eta \right]}_{\mathrm{avg}}}^{2}\times \sqrt{2\times {\left(\frac{\sigma {\left[\eta \right]}_{\mathrm{avg}}}{{\left[\eta \right]}_{\mathrm{avg}}}\right)}^{2}}$, x the slope determined from linear regression, and σx error of the slope.

An exponential coefficient (*k*_*exp*_) for the relative viscosity was calculated from fitting $\eta_{\mathrm{rel}}$ to Equation 10 with the Generalized Reduced gradient least squares solver function. This empirical model has previously been applied to systems in which an exponential relationship exists between viscosity and concentration:^26,33^

(10) $$\eta_{\mathrm{rel}}=e^{k_{\exp}\times c}$$

#### Theoretical hydrodynamic properties

Hydrodynamic volume fraction and predicted intrinsic viscosities were computed for each anti-IL-8 molecule. The volume fraction (*ϕ*) per mutant was computed as follows:^34^

$$r_{h}={\left(\frac{3\left[\eta \right]M_{w}}{10\pi N_{A}}\right)}^{\frac{1}{3}}$$

$$\phi =\left(\frac{cN_{A}}{M_{w}}\right)\frac{4\pi r_{h}}{3}$$

where $r_{h}$ is the hydrodynamic radius (nm) determined from *[η]* (nm^3^/g), Mw is molecular weight (g/mol), and *N*_*A*_ is the Avogadro constant (mol^−1^). This was used to calculate *ϕ* (the volume fraction (nm^3^/g)), at a certain *c* (concentration (g/nm^3^)).

#### Hydrodynamic properties predictions

The HYDROPRO program (version 10)^35^ was used to predict the hydrodynamic properties for each anti-IL-8 molecule. Full IgG homology constructs were exported in pdb format and analyzed using the residue-level shell and bead calculation modes, with partial specific volumes set at 0.75 mL. Molecular weights were obtained from peptide mapping liquid chromatography-mass spectrometry experiments.^19^

#### Shape factor estimation

The shape factor (*v*) was calculated by computing the solvent-accessible surface area (*SASA*) and protein volume (water probe) of full IgG homology constructs generated in MOE (Chemical Computing Group, Montreal, Canada)^36:^

$$\Psi =\frac{\mathrm{SASA}}{V^{2/3}}$$

Ψ=1.454v+7.085

where *Ψ* is the geometric shape coefficient and v the shape factor.

## Biophysical analysis of anti-IL-8 molecules

### Assessment of colloidal parameters

Dynamic light scattering was used to measure diffusion coefficients in the 1–20 mg/mL concentration range (Stunner, Unchained Labs, CA, USA).^19^ Diffusion coefficients and size data were calculated from the correlation function, which uses cumulant analysis within the Stunner software (Unchained Labs, CA, USA). All measurements had cumulative variance <0.01 and correlogram amplitude intercepts of >0.6. Formulation buffer alone measurements showed no significant decay in correlograms over experiment duration. Hydrodynamic radii (r_hDLS_) were obtained by halving the mean Z-ave diameters obtained for each molecule. Five measurements were performed at 25°C with 10-s acquisition times. A 1% extinction coefficient of 1.55 AUL/(gcm) was applied for all panel molecules. Samples were prepared in formulation buffer (pH 6.0) and data were analyzed using the Lunatic & Stunner software (Unchained Labs, CA, US, version 8.1.0.244). The self-interaction parameter, *k*_*D*_, was obtained from the linear fit of concentration-dependent diffusion coefficient behavior:

$$D_{\mathrm{app}}=D_{0}\left(1+k_{D}c\right)$$

where *D*_*app*_ is the cumulative diffusion coefficient, *D*_*0*_ the self-diffusion coefficient at infinite dilution, and *k*_*D*_ the interaction parameter. This equation assumes linearity of diffusion coefficients over a concentration range (*c*), which is usually valid for antibody solutions at <10 mg/mL concentrations.

### Measurement of isoelectric point

Capillary isoelectric focussing was used to measure charge heterogeneity and isoelectric point of the IgG panel, using a previously described method.^19^ The Empower 3 software (v4, Waters, US) was used for data processing, isoform assignment, and analysis.

### Electrophoretic light scattering

The zeta potential (*ζ*) of all molecules was measured in the formulation buffer (pH 6.0, 5 mg/mL) using a Zetasizer Nano ZS (Malvern Panalytical, Malvern, UK).^19^ All samples were filtered before measurements that used default settings; an equilibration time of 120 s, automatic attenuation, and 10–100 measurement runs with a one-minute pause between measurements. At least three technical replicate measurements were performed and standard deviations are reported.

### Statistical analysis

JMP Pro (version 17.0.0, 2022) was used for correlation analysis between computational and experimental data and predictive modeling. For partial least squares modeling (PLS), a non-linear iterative PLS algorithm was selected with leave one-out cross-validation. This model is based on the lowest mean root predicted residual error sum of squares (PRESS) and the best Q^2^ (i.e. 1 − PRESS/sum of squared deviation from mean). Training data included all panel IgGs and the test data consisted of four in-house early-stage molecules.

## Results

In this study, we applied various viscosity modeling approaches to predict and fit concentration-dependent viscosity profiles of a panel of nine anti-IL-8 IgG1 molecules formulated in the same buffer. Eight of these IgGs carry single-point mutations, designed to disrupt negative (D17N, D70N, D28N, and D56N), hydrophobic (V5Q, W32Q), or positive patches (K42E, R53G). Computational and experimental molecular descriptors for this IgG panel are published elsewhere.^19^ We examined the solution viscosity at high (≤120 mg/mL) and ultra-high (≤260 or noted as >120 mg/mLmg/mL) mAb concentrations to compare viscosity–concentration profile fits.^1^

### Viscosity profile fitting for the anti-IL-8 mAb mutant panel

**Figure 1.**
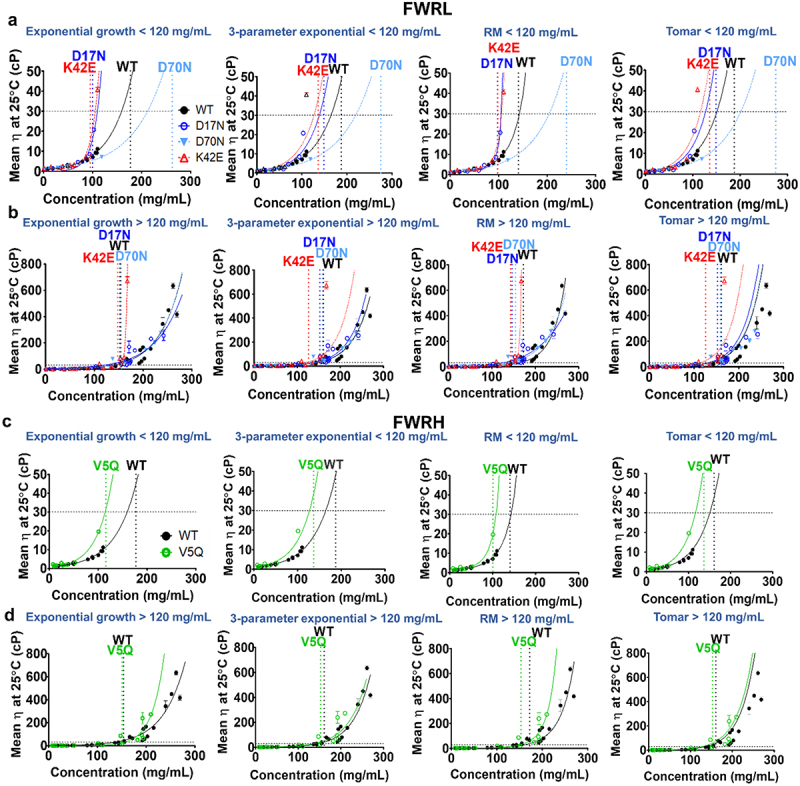
Anti-IL-8 IgG framework mutant concentration–viscosity profiles were fitted with four models; an exponential growth equation, a three-parameter exponential model, a modified Ross-Minton (RM) model and the Tomar model (left to right). A horizontal dotted line at 30 cP is the threshold for ‘acceptable’ viscosity. Vertical dotted lines for each molecule mark the ‘knee’ of each viscosity–concentration curve. FWRL (a-b) and FWRH (c-d) mutants viscosity data in two concentration regimes are shown; ≤ 120 mg/mL (top row) and > 120 mg/mL (bottom row). Error bars represent standard deviation (*N*=2). Abbreviations: FWRL= light chain framework region, FWRH= heavy chain framework region, RM= Ross-Minton model.

**Figure 2.**
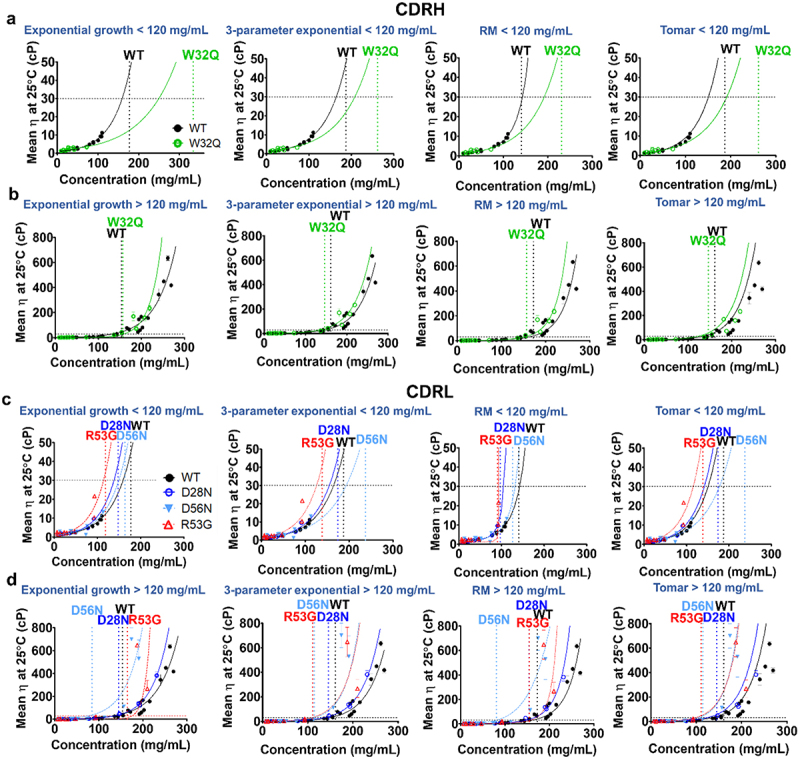
Anti-IL-8 CDR mutant concentration–viscosity profiles were fitted with four models; an exponential growth equation, a three-parameter exponential model, a modified Ross-Minton (RM) model and the Tomar model (left to right). A horizontal dotted line at 30 cP is the threshold for ‘acceptable’ viscosity. Vertical dotted lines for each molecule mark the ‘knee’ of each viscosity–concentration curve. CDRH (a-b) and CDRL (c-d) viscosity data in two concentration regimes are shown; up to 120 mg/mL (top row) and over 120 mg/mL (bottom row). Error bars show standard deviations per viscosity measurement (*N*=2). Abbreviations: CDRL= light chain complementarity determining region, CDRH= heavy complementarity determining region, RM= Ross-Minton model.

Viscosity–concentration data are crucial for fitting models that extrapolate to higher concentrations or interpolate between points. Table 2 compares four viscosity–concentration fit models across two regimes: ≤120 mg/mL (high-concentration) and ≤260 mg/mL (ultra-high concentration) (Figures 1 and 2) formulated in the same buffer (pH 6.0). While D70N and W32Q mutants exhibited viscosity reduction relative to the WT at the high-concentration regime (Figures 1a and2a), none exhibited reduced viscosity at ultra-high mAb concentrations.

**Table 2.** **Model results of interpolated/extrapolated viscosity values at 180 mg/mL and predictions for ultra-high concentration viscosities (at 180 mg/mL) by models trained from high concentration data only**. a, results from growth exponential and three-parameter exponential models. b, results from modified Ross Minton and Tomar equation models. Coefficient of determination and goodness-of-fit (R^2^ parameter). *Predicted viscosity is >20,000 cP or has reached model failure (~0 cP). 95% confidence intervals are shown in brackets below calculated viscosities. Percentage differences between viscosity predictions at 180 mg/mL from ultra-high-concentration versus high-concentration data were calculated with $\frac{c_{\mathrm{HIGH}}-c_{\mathrm{MID})}}{\begin{array}{c} \frac{c_{\mathrm{HIGH}}+C_{\mathrm{MID}}}{2} \end{array}}*100$. Viscosity predictions from the high-concentration data that exceeded predictions from the ultra-high-concentration data (negative % differences) are highlighted in red.

		Growth exponential					3-parameter exponential				
	a	Model results			High concentration model prediction		Model results			High concentration model prediction	
Molecule	Concentration regime	Viscosity modelled (cP) at 180 mg/mL	R^2^	% difference in η between conc. regime	Viscosity prediction (at 180 mg/mL) (cP)	R^2^	Viscosity modelled (cP) at 180 mg/mL	R^2^	% difference in η between conc. regime	Viscosity prediction (at 180 mg/mL) (cP)	R^2^
WT	<120 mg/mL (high)	47.69 (43.12–51.9)	0.975	52%	47.69 (34.77– 54.97)	0.911	41.23 (37.7–44.76)	0.974	45%	41.23 (33.57–48.89)	0.912
	>120 mg/mL (ultra-high)	81.24 (65.36–97.12)	0.910				65.29 (48.78–81.80)	0.905			
D17N (FWRL)	<120 mg/mL (high)	2442.88 (1831.02–3054.74)	0.958	–186%	2442.88 (–2.85x10^5^–2.89x10^5^)	0.482	86.43 (64.72–108.14)	0.848	–9%	86.43 (63.43–109.43)	0.762
	>120 mg/mL (ultra-high)	90.89 (76.96–104.82)	0.817				79.34 (59.58–99.1)	0.767			
D70N (FWRL)	<120 mg/mL (high)	18.26 (17.32–19.2)	0.998	124%	18.26 (16.4–20.12)	0.955	16.8 (15.86–17.74)	0.997	122%	16.8 (15.07–18.53)	0.951
	>120 mg/mL (ultra-high)	77.52 (69.4–85.64)	0.973				69.24 (61.26–77.22)	0.972			
K42E (FWRL)	<120 mg/mL (high)	4228.5 (3803.84–4653.16)	0.993	120%	4228.5 (–519.66–8976.66)	0.718	121.63 (99.83–143.43)	0.862	31%	121.63 (106.94–136.32)	0.472
	>120 mg/mL (ultra-high)	17023.12 (15575.1–18471.2)	0.984				166.9 (146.25–187.55)	0.496			
V5Q(FWRH)	<120 mg/mL (high)	245.03 (226.15–281.91)	0.985	101%	245.03 (119.65–370.41)	0.779	44.54 (40.19–48.89)	0.974	57%	44.54 (21.26–65.80)	0.762
	>120 mg/mL (ultra-high)	80.76 (60.84–100.68)	0.789				80.42 (65.63–95.21)	0.754			
W32Q (CDRH)	<120 mg/mL (high)	13.02 (8.81–17.23)	0.552	132%	13.02 (10.49–15.55)	0.743	19.62 (11–28.24)	0.538	130%	19.62 (15.61–23.63)	0.807
	>120 mg/mL (ultra-high)	63.16 (49.04–77.28)	0.986				92.87 (74.24–111.5)	0.947			
D28N (CDRL)	<120 mg/mL (high)	89.38 (73.49–105.27)	0.942	7%	89.38 (86.12–92.64)	0.997	51.77 (43.62–59.92)	0.927	59%	51.77 (49.07–54.47)	0.989
	>120 mg/mL (ultra-high)	95.98 (92.37–99.59)	0.997				94.77 (91.2–98.34)	0.997			
D56N (CDRL)	<120 mg/mL (high)	62.2 (46.11–78.29)	0.841	154%	62.2 (56.22–68.18)	0.817	24.08 (20.26–27.9)	0.791	167%	24.08 (21.76–26.4)	0.800
	>120 mg/mL (ultra-high)	481.55 (385.52–577.58)	0.813				263.87 (228.28–299.46)	0.802			
R53G (CDRL)	<120 mg/mL (high)	208.26 (143.97–272.55)	0.825	–134%	208.26 (138.55–277.97)	0.835	113.15 (85.43–140.45)	0.819	82%	113.15 (79.33–146.97)	0.808
	>120 mg/mL (ultra-high)	41.26 (–72.12–154.64)	0.920				269.73 (203.71–389.75)	0.840			
		Modified Ross-Minton					Tomar				
	b	Model results			High concentration model prediction		Model results			High concentration model prediction	
Molecule	Concentration regime	Viscosity modelled (cP) at 180 mg/mL	R^2^	% difference in η between conc. regime	Viscosity prediction (at 180 mg/mL) (cP)	R^2^	Viscosity modelled (cP) at 180 mg/mL	R^2^	% difference in η between conc. regime	Viscosity prediction (at 180 mg/mL) (cP)	R^2^
WT	<120 mg/mL (high)	154.59 (141.85–167.33)	0.982	–106%	154.59 (–5.82x10^4^–5.85x10^4^)	0.33	59.4 (53.31–65.49)	0.977	53%	59.4 (45.66–73.14)	0.908
	>120 mg/mL (ultra-high)	47.63 (25.38–69.88)	0.865				102.5 (66.85–138.15)	0.892			
D17N (FWRL)	<120 mg/mL (high)	Too high*	0.991	–	Overpredictive	–	139.72 (96.5–182.94)	0.866	–10%	139.72 (72.37–207.07)	0.727
	>120 mg/mL (ultra-high)	94.22(80.22–108.22)	0.826				126.74 (73.36–180.12)	0.733			
D70N (FWRL)	<120 mg/mL (high)	19.76 (18.93–20.59	0.999	119%	19.76 (17.79–21.73)	0.97	20.97 (20.08–21.86)	0.999	135%	20.97 (13.28–28.66)	0.962
	>120 mg/mL (ultra-high)	77.88 (69.74–86.02)	0.973				107.72 (90.33–125.11)	0.967			
K42E (FWRL)	<120 mg/mL (high)	Too high*	0.999	–	Overpredictive	–	199.84(155.92–243.76)	0.887	38%	199.84 (173.2–226.5)	0.499
	>120 mg/mL (ultra-high)	Too high*	0.990				294.29(246.69–341.89)	0.524			
V5Q (FWRH)	<120 mg/mL (high)	Too high*	0.993	–	Overpredictive	–	193.43 (171.29–215.57)	0.981	–43%	193.43(119.49–267.37)	0.773
	>120 mg/mL (ultra-high)	79.76 (60.35–99.17)	0.795				124.59 (94.07–155.11)	0.766			
W32Q (CDRH)	<120 mg/mL (high)	24.49 (12.52–36.46)	0.527	96%	24.49 (19.39–29.59)	0.85	24.67 (12.32–36.66)	0.528	143%	24.67 (19.54–29.8)	0.843
	>120 mg/mL (ultra-high)	70.02 (56.39–83.65)	0.989				148.81 (118.65–178.97)	0.967			
D28N (CDRL)	<120 mg/mL (high)	Too high*	0.991	–	Overpredictive	–	76.42 (62.88–89.96)	0.937	67%	76.42 (73.45–79.39)	0.996
	>120 mg/mL (ultra-high)	76.21 (66.69–85.73)	0.982				153.64 (143.25–164.03)	0.996			
D56N (CDRL)	<120 mg/mL (high)	892.43(675.94–1108.92)	0.878	–58%	892.43 (–-543.9–2328.8)	0.46	31.2 (25.48–36.92)	0.807	177%	31.2 (28.2–34.2)	0.809
	>120 mg/mL (ultra-high)	491.72(391.07–592.37)	0.824				503.71 (377.53–629.89)	0.787			
R53G (CDRL)	<120 mg/mL (high)	Too high*	0.995	–	Overpredictive	–	178.38 (124.89–231.87)	0.823	93%	178.38(121.11–235.65)	0.827
	>120 mg/mL (ultra-high)	79.43 (–24.96–183.82)	0.930				486.36 (282.4–690.32)	0.857

A critical feature of viscosity–concentration curves is the identification of the point where the curvature shifts to pseudo-exponential growth. We used this knee point to define this critical concentration and compared this knee point in four model fits derived from the same viscosity–concentration data in two concentration regimes for all nine molecules. Significant shifts in the knee of viscosity–concentration curves were observed with each model, altering conclusions about the viscosity-altering effects of single-point mutations (Table 1). For example, D70N consistently showed reduced viscosity relative to WT in all fits at the lower concentration regime (Figure 1a). However, when including ultra-high concentration data, overall viscosity reduction was negligible except with the exponential growth model (Figure 1b). These findings demonstrate the importance of capturing complete viscosity–concentration profiles rather than relying on interpolated or extrapolated data, as conclusions may vary considerably between different viscosity models and concentration regimes.

The Ross-Minton model describes the self-crowding parameter, *k*, and Simha shape parameter, *v*. Previous work has reported non-self-associating mAbs to have a *k/v* value of 0.38.^31^ Here, we observed much lower *k/v* values, indicating an increase in molecular packing resulting from increased attractive forces, particularly at the high concentration (>120 mg/mL) regime viscosity profiles (Supplementary Table S2).

Goodness-of-fit assessment (average R^2^) for the growth-exponential or modified Ross-Minton models across both concentration regimes were determined as 0.90 and 0.92 for high- and ultra-high-concentration range data, respectively (Supplementary Information). However, both the growth-exponential and Ross-Minton equations significantly overestimated predicted viscosity with the high-concentration range viscosity–concentration data (Table 2).

A key question to answer is if the models from high concentration data can predict or scale to ultra-high concentration data (Table 2 and Supplementary Information)? Large discrepancies in the predicted viscosity from the high concentration regime to the ultra-high concentration regime were observed (% differences −186% to + 177%), highlighting the lack of predictive power from dose-relevant ultra-high concentrations based on viscosity data obtained for the high-concentration regime. Additionally, comparisons of the ranking of predicted viscosities per molecule from the high concentration at 180 mg/mL did not align to the ultra-high concentration regime for any of the model equation fits (Table S3). Most predictions of ultra-high concentration data based on the high-concentration regime viscosity models demonstrated increased uncertainty and error, as well as reduced accuracy in measured viscosity points. For example, the growth exponential model for V5Q showed increased uncertainty when predicting ultra-high concentration viscosities (180 mg/mL) with broadening of 95% confidence intervals (from predicted 245 (±27.8) cP to 245 (±125) cP) and reduced R^2^ (from 0.98 to 0.78). Additionally, the modified Ross-Minton high concentration viscosity models showed numerous overpredictions. High concentration viscosity measurements may not accurately account for the complex anisotropic interactions occurring at ultra-high mAb concentrations with increased molecular crowding, including the formation of clusters or transient networks.^12,37^

### Intrinsic viscosity, pair-wise interactions, and hydrodynamic properties of the anti-IL-8 mAb panel

One method of assessing anti-IL-8 mAb contribution to the solution viscosity is to determine the intrinsic viscosity (*[η]*) (Supplementary Table S4). Apparent dynamic viscosity was calculated in the dilute concentration regime (0–50 mg/mL), and reduced viscosities (*η*_*red*_) and *ln(η*_*rel*_*)/c* plotted against mAb concentration (Supplementary Figures S1 and S2). The intercept of each plot was reported as intrinsic viscosity, *[η]*_*H*_ and *[η]*_*K*,_ which were averaged to determine *[η]*_*avg*_. Most anti-IL-8 mAb mutants had similar *[η]*_*avg*_ values, but R53G had a higher intrinsic viscosity, indicating an increased excluded volume effect.

To account for any curvature in reduced viscosities over concentration plots, a second order polynomial fit was applied to the *η*_*rel*_ profiles of each anti-IL-8 mAb (Supplementary Figure S3), with the assumed approximation that intrinsic viscosity (*[η]*_*v*_)= *k*_*1*_ in the equation $\eta_{\mathrm{rel}}=1+k_{1}c+k_{2}c^{2}$.^38^ The *[η]*_*v*_ values aligned with linear-derived intrinsic viscosity (*[η]*_*avg*_) (R^2^ = 0.73) (Supplementary Figure S4), but showed increased variation amongst the anti-IL-8 panel.

The HYDROPRO tool^39^ was used to compute the intrinsic viscosity (*[η]*_*HYD*_) and radius of gyration (*R*_*g*_) estimations for anti-IL-8 mutants (Supplementary Table S4). These were computed at residue levels in both shell and bead mode. Though no strong correlations were found with *[η]*_*HYD*_ to experimental *[η]* (<0.7 R^2^) in either mode, the residue-shell mode better aligned to the experimental intrinsic viscosity (*[η]*_*avg*_) (R^2^ = 0.59) than the residue-bead mode (R^2^ = 0.20) (Supplementary Figure S5).

Intrinsic viscosity can also be derived from the Ross-Minton model (Table 1 and Supplementary Table S2). Here, the Ross-Minton derived viscosities poorly correlated with *[η]*_*avg*_ in both concentration regimes (Supplementary Figure S6). This demonstrates how the ultra-high-concentration data, from which the Ross-Minton viscosity is derived, skews *[η]* to no longer be representative of molecular size and molecular diffusivity in the ultra-dilute regime.

Next, we compared the linear correlations of hydrodynamic radius (*r*_*h*_) measurements derived from the linear intrinsic viscosity (*[η]*_*avg*_) and polynomial intrinsic viscosity (*[η]*_*v*_) to the dynamic light scattering (DLS)-derived hydrodynamic radius (*r*_*hDLS*_) (Figure 4). R53G, which had the highest *r*_*h*_ from intrinsic viscosity, did not exhibit an increase in hydrodynamic size measured by DLS, resulting in poor correlation with the intrinsic viscosity-derived rh (Figure 4a) which was influenced from the high intrinsic viscosity of R53G. However, the use of *[η]*_*v*_ to derive *r*_*h*_ (*rh*_*[η]v*_) resulted in no correlation to *r*_*hDLS*_ (Figure 4b). This indicates potential inaccuracies in Z-ave DLS measurements at 1 mg/mL and misrepresentation of intrinsic viscosity from using the polynomial function on *η*_*rel*,_ when accounting for higher-order interactions. The hydrodynamic radii obtained from polynomial fitting resulted in physically unrealistic values (3–9 nm) and therefore should be discounted. Effective volume fraction (*ϕ*) was computed up to 100 mg/mL from *[η]*_*avg*_ according to Equation 11B (Figure 4c). R53G exhibited a significantly higher hydrodynamic volume across the whole concentration range.

Two methods were used to generate shape information for each anti-IL-8 molecule (Supplementary Figure S7). The HYDROPRO program was used in shell mode to predict the radius of gyration (*R*_*g*_) and its ratio to *r*_*h*_ (the shape ratio (*ρ*)) was computed for each molecule (Supplementary Figure S7a). We used *r*_*h*_ values from both intrinsic viscosity (linear (*[η]*_*avg*_) and polynomial (*[η]*_*v*_) and DLS. The shape ratio determined for each molecule was ~0.775, which was attributed to spherical, globular proteins.^40–42^ Interestingly, R53G showed the lowest ratios *R*_*g*_*/r*_*h[η]avg*_ and *R*_*g*_*/r*_*h[η]v*_ demonstrating a deviation in shape.^43,44^ Use of DLS *r*_*h*_ values showed comparable ratios for all anti-IL-8 molecules.

Subsequently, shape coefficients (*Ψ*) and shape factors (*v*) were calculated from computed solvent-accessible surface areas (*SASA*) and protein volumes of IgG homology constructs of the anti-IL-8 panel (Supplementary Figure S7b). Since the same IgG1 scaffold was used with single amino acid substitutions for the anti-IL-8 panel, *SASA* and protein volumes were comparable, and therefore no distinguishable differences in shape factors were observed.

### Computational viscosity predictions of the anti-IL-8 mAb mutant panel

Numerous empirical viscosity prediction models from small datasets of proprietary development phase mAbs have been developed to date. Li et al. studied the relationship of 18 different molecular descriptors on 11 Fv constructs to viscosity data of these mAbs at 150 mg/mL.^13^ The best regression model for viscosity prediction was found to include isoelectric point and aggregation propensity predictions (Equation 1). A similar regression model on 14 IgG1 mAbs viscosity data at 180 mg/mL was developed by Sharma et al., drawing a relationship between viscosity and Fv charge, charge symmetry, and hydrophobic index (Equation 2).^14^ Finally, Tomar et al. found that pI correlated with parameter B in the logarithmic fitting of 16 development-phase mAb viscosity profiles at concentrations up to 180 mg/mL (Equation 3A). Here, we used these approaches to derive viscosity prediction scores for an anti-IL-8 mAb panel (Figure 6). With all models, negative patch disrupting mutants were predicted to have lower viscosity compared to the WT, with ≤30 cP predicted for mutants (180 mg/mL) using the Sharma scores. Positive patch disrupting mutants were predicted to increase viscosity compared to WT IgG, with viscosities >30 cP with both Sharma and Tomar scores at 180 mg/mL. Hydrophobic patch disrupting mutants showed similar predicted viscosities to WT as these models primarily use charge-based descriptors. With regards to model performance, the Li viscosity model significantly underestimates viscosities at 150 mg/mL when comparing to all experimental model fits (Figure 5b). For viscosity predictions at 180 mg/mL, the true experimental viscosity appears to lie in between the Sharma and Tomar viscosity scores (Figure 5e), with Sharma scores underpredicting and Tomar scores overpredicting viscosities. Since inaccuracies from quantitative predictions from the Li, Sharma, and Tomar scores were observed, we next assessed qualitative prediction to identify if molecule rankings are correct (Supplementary Table S5). None of the predictions directly matched the experimental viscosity ranking of the anti-IL-8 panel.

A previous study by Kingsbury et al. examined a larger dataset (*N* = 59) that included approved ‘developable’ mAbs to better identify non-redundant *in silico* descriptors and experimental parameters that result in improved developability characteristics (viscosity and opalescence).^45^ Clustering analysis showed distinct correlations between mAbs with favorable developability characteristics and *k*_*D*_, measured pI and the effective charge. They also assessed sequence-based molecular descriptors (hydrophobic index and charge symmetry), finding that such singular descriptors did not have the same discrimination level as the experimental parameters.

In our study, we used the same parameters and thresholds to test these trends to viscosity, using both high and ultra-high concentration data (Figure 6 a,b respectively). Here, we could not clearly distinguish between low or high viscosity molecules at either concentration regime examined with use of either experimental parameters (*k*_*D*_, pI or zeta potential) or molecular descriptors.

The structure-based ensemble charge (ens_charge) descriptor has previously been shown to correlate with viscosity; molecules with ens_charge values of ≥+2 C were correlated with reduced viscosity.^46^ Here, only positive patch disrupting mutants had an ens_charge of <+2 (Supplementary Table S1). At 120 mg/mL, these mutants had high viscosities (>30 cP), but the three highly viscous molecules had ens_charge values >+2 (false positives) when examining the high concentration data (67% accuracy) (Supplementary Table S8), suggesting limitations in the use of this singular descriptor for predicting viscosity. Interpolations from ultra-high concentration data for viscosity at 120 mg/mL resulted in 55% accuracy, with both false negatives and false positives present.

### Predictive modelling of the anti-IL-8 molecule panel

We next sought to develop and assess a simple regression model to predict the viscosity of the anti-IL-8 panel (Figure 7). Here, we averaged the ‘knee’ found for each model-fitted viscosity profile and assessed correlations with computational molecular descriptors (Supplementary Figure S10). Five of the highest correlating variables (R > 0.5) were used in PLS regression, resulting in a final equation (Figure 7b) with an R^2^ value of 0.76 (Figure 7d). Model performance was tested with a set of four in-house molecules (Ab1–4), which saw a reduction in R^2^ to 0.31 (Figure 7e). The regression model underpredicted the viscosity knee values for the test molecules, which were significantly higher than the training anti-IL-8 panel, demonstrating the need for a case-by-case approach when predicting viscosity, particularly with small datasets.

## Discussion

Reducing high solution viscosity in concentrated mAb formulations is a crucial aim in mAb drug development, achievable through sequence-based^30,47,48^ and formulation strategies.^2,49,50^ Predicting sequences that lead to elevated viscosity at high mAb concentrations can aid in early-stage candidate selection and risk assessment.

In this work, we used structure- and sequence-based molecular descriptors to compare our predictions with experimentally derived viscosity parameters across different concentration regimes. Our focus was on evaluating viscosity predictions from model fits and hydrodynamic properties, as well as from empirical and machine-learning derived models for a panel of anti-IL-8 IgG molecules. These molecules include single-point mutations designed to target electrostatic or hydrophobic surface patches, known to significantly impact viscosity.^19^

### Interpretation of viscosity data dependent on concentration regime and model fit and lack of predictive power from high concentration models to ultra-high concentration viscosities

Routes of administration with volume limitations, such as subcutaneous injection, require high (>100 mg/mL) or ultra-high (>150 mg/mL) mAb concentrations to achieve effective therapeutic doses. In this study, we defined a high concentration regime up to 120 mg/mL and an ultra-high concentration regime up to 260 mg/mL. The viscosity of mAb formulations at these concentrations is influenced not only by intrinsic factors like hydrodynamic size, anisotropic surface charges, and hydrophobicity, but also by concentration-dependent effects.^9,48,51,52^ Contributions to viscosity present in infinitely dilute systems are typically characterized using light scattering techniques to determine the self-interaction parameter, *k*_*D*_, or the second osmotic virial coefficient, *B*_*22*_. However, in more concentrated solutions - involving pairwise and higher-order associations- the interactions become more complex and challenging to model.

We assessed the viscosity profiles of an anti-IL-8 mutant panel in two concentration regimes (high and ultra-high) (Figures 1 and 2). While two mutants (D70N and W32Q) initially exhibited reduced viscosity compared to the wild-type (WT) molecule at lower concentrations, including ultra-high concentration data revealed that all mutants exhibited higher viscosities relative to WT. Therefore, all anti-IL-8 molecules were classified as ‘high risk’ in terms of their developability at clinically relevant concentrations.

This study represents the first direct comparison of multiple viscosity fitting models and their impact on data interpretation. We selected four prominent models routinely used for viscosity curve analysis (Table 1). Our findings indicate that the exponential growth or modified Ross Minton equations are optimum models, showing the highest R^2^ values. However, extrapolating viscosity from lower concentration data to higher concentrations, where higher-order interactions introduce complexities, proved challenging (Table 2). Additionally, we propose using the ‘knee’ of each curve to standardize the point where viscosity begins to increase exponentially, facilitating comparison between models. The notable differences in modeled viscosities at identical concentrations emphasize the importance of examining complete concentration–viscosity profiles rather than relying on interpolated or extrapolated results.

We also assessed parameters derived from the modified Ross-Minton model fit, such as the *k/v* crowding factor and intrinsic viscosity (*[η]*_*RM*_). We observed increased molecular packing and attractive forces in the ultra-high concentration regime, represented with lower *k/v* values (Suplementary Table S2). However, *[η]*_*RM*_ did not align with experimental *[η]* values (Supplementary Figure S6), suggesting overfitting of high concentration viscosity data using the generalized reduced gradient algorithm, and highlighting the lack of translatability between concentration regimes (experimental *[η]* measured in 0–50 mg/mL range).

This lack of translatability was further exemplified by assessing accuracy and errors of high-concentration models generated for each anti-IL-8 molecule to predict ultra-high concentration data (Table 2). Molecule rankings for viscosity prediction at 180 mg/mL showed misalignment between concentration regimes across all model equation fits adopted (Table S3). The increased uncertainties of the models and reduced alignment to ultra-high concentration data supported how different mechanisms which may be contributing to viscosity prevail as mAb concentration increases.

### Viscosity predictions and deeper mechanistic understanding from hydrodynamic parameters

Traditionally, the hydrodynamic behavior of proteins has been modeled from colloidal principles and expanded with the integration of polymer science to account for shape anisotropy.^53^ Here, we assessed the anti-IL-8 molecule panel with an extensive range of hydrodynamic parameters, such as intrinsic viscosity (*[η])*, the Huggins (*k*_*H*_) coefficient, shape descriptors, and volume fraction. Our goal was to identify correlations between high concentration viscosities and other previously determined biophysical parameters.^19^

**Figure 3.**
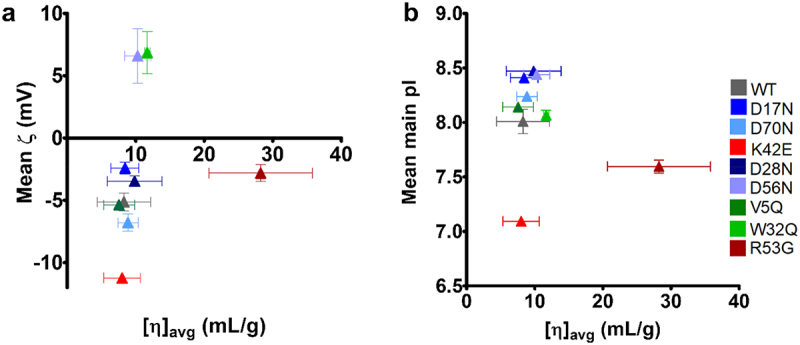
No correlation between average intrinsic viscosity and anti-IL-8 mAb charge parameters. No strong correlations were observed between [η]_avg_ and charge parameters, zeta potential (ζ) (a) or mean isoelectric point (pI) (b). Vertical error bars represent standard deviations for ζ and pI. Horizontal error bars represent the standard error of [η]_avg_. Logarithmic equations shown for k_H_/k_K_ over [η]_avg_ plot.

**Figure 4.**
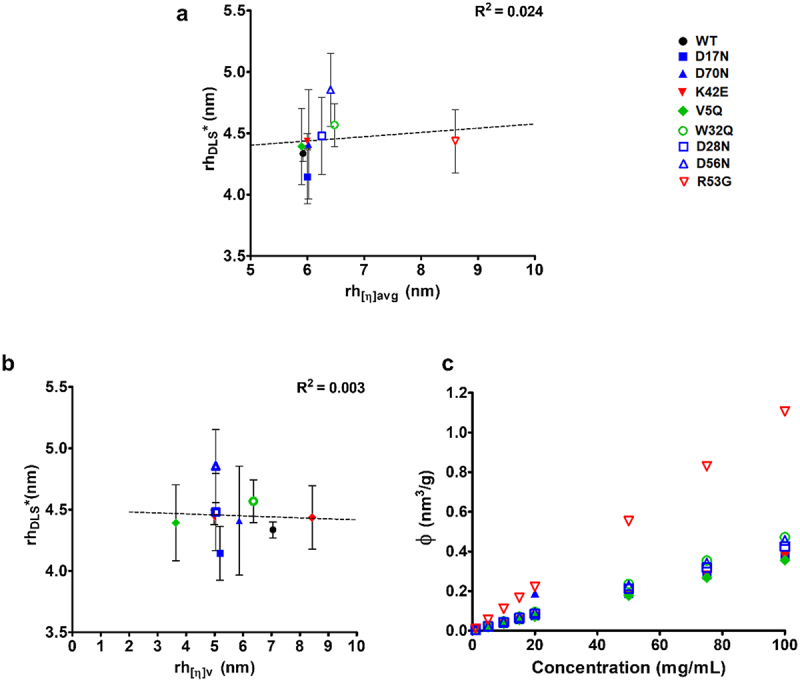
Hydrodynamic diameter from intrinsic viscosity versus dynamic light scattering and effective volume fraction of anti-IL-8 molecules. the hydrodynamic radius (*r_h_*) of each molecule at 1 mg/mL was derived either from intrinsic viscosity measurements with **equation 12A** or from dynamic light scattering (DLS) Z-ave measurements. DLS-derived *r_h_* values poorly correlated to *r_h_* values from either the r_h_ derived from average intrinsic viscosity (r_h[η]avg_) (**a**), or from polynomial intrinsic viscosity (r_h[η]v_) (b). r_h[η]avg_ was used to compute the theoretical effective volume fraction (ϕ) for each molecule up to 100 mg/mL(c).

For most mutants, *[η]* values were comparable to the WT molecule (Suplementary Table S2 and Figure 3), indicating similar contributions from their respective hydrodynamic sizes, as illustrated by the computed volume fraction occupied (*ϕ*) (Figure 4). This was unsurprising as we hypothesized changes in excluded volume would unlikely result from single-point mutations. However, the R53G mutant, which disrupts positive patches, exhibited increased viscosity and inferred size, although this was not consistently reflected in Z-ave data from DLS measurements at 1 mg/mL, affecting the correlation between hydrodynamic radius (*r*_*h*_) derived from viscosity (*[η])* and *r*_*h*_ derived from DLS (Figure 4a,b). It is worthwhile noting that the Z-ave data at such low mAb concentrations may be more significantly impacted by the presence of trehalose in the formulation buffer, with preferential exclusion of the sugar to the mAb surface, reducing the protein-water interfacial area and affecting apparent hydrodynamic size.^54,55^ Beyond concentration and formulation composition dependence, size data from DLS also assume molecule sphericity, are orientation dependent and can have calculation inaccuracies with solutions that have increased polydispersity, depending on the mathematical algorithm used to compute diffusivity and estimate size. Pair-wise interaction quantification from *k*_*H*_ values showed consistent results for most molecules, except for W32Q and notably R53G, which exhibited lower inferred pair-wise contributions (Supplementary Table S4). These findings align with the viscosity profiles observed for W32Q (hydrophobic patch-disrupting mutant) in the high concentration regime (Figure 2a), but contrast with the ultra-high concentration viscosity (Figure 2c,d) and increased *[η]* was observed for R53G. Furthermore, unlike other mutants, R53G *η*_*red*_ does not scale linearly with concentration (Suplementary Figure S1).

We hypothesize that this mutant forms clusters starting from concentrations as low as 1 mg/mL, which may explain why differences in *rh*_*DLS*_ were not detected. In these small clusters, short-ranged attractive interactions primarily affect neighboring molecules interacting in the cluster. However, long-range repulsive interactions are additive and apply in an isotropic range from the cluster.^33,56^ This could result in an effect where the cluster stabilizes at a critical cluster number, as the attractive forces remain in play due to the reduced distance between the molecules but the combined repulsion repels any new entrants to the cluster. When measuring interactions based on bulk solution flow properties, the base unit being measured would be the cluster as the constituent proteins move as one entity within the flow. The cluster has a larger hydrodynamic radius than the underlying mAb leading to the observed increased intrinsic viscosity, however, the clusters do not interact strongly with one another due to resulting in the low observed *k*_*H*_. The base unit being measured in DLS is the protein, even when clustering is occurring, demonstrated by the more negative *k*_*D*_ for R53G observed in the 1–30 mg/mL range, stemming from the reduced molecular diffusivity and increased self-association of the protein when entrained in a cluster. Conversely, W32Q had a less negative *k*_*D*_, aligning with reduced *k*_*H*_. Further size and structural analysis for R53G, such as assessing R_g_ experimentally with multi-angle or X-ray scattering, would confirm this clustering hypothesis.

It is worthwhile to note that the variability in both concentration and viscosity measurements results in large measurement errors for intrinsic viscosity. Furthermore, the derivation of intrinsic viscosity from linear regression of solutions exceeding the infinitely dilute regime and containing higher-order interactions, results in the misrepresentation of pair-wise contributions. Previous work has demonstrated minimum and maximum concentration limits for protein solutions, around 2–40 mg/mL, observing a three-state power law model for log(η) versus log(c).^38^ To reduce the curvature of our data, we excluded the highest concentration data points for each molecule in the *η*_*red*_*/c* plots. Overall, this approach did not significantly reduce *[η]* error, and in most cases the linear fit was dependent on only three data points (Suplementary Figure S2). We therefore chose to include the polynomial fitting of η_rel_ over concentration to derive [η]_v,_ previously proposed by Yadav and coworkers,^51^ but this similarly showed high error (Suplementary Figure S3).

We examined correlations of multiple biophysical parameters to the hydrodynamic properties and viscosity of the anti-IL-8 molecule panel (Supplementary Table S5). According to the primary electroviscous effect, the higher the net charge of a molecule, the greater the distortion of the electrical double-layer (EDL) surrounding the molecule, increasing drag force in solution and *[η]*.^57^ Here, we saw no correlation in surface zeta potential (*ζ*) nor isoelectric point to *[η]* for the anti-IL-8 molecules (Figure 3). This lack of correlation has been observed previously^38,51^ and suggests that *ζ* measurements at 5 mg/mL (where different molecular weight species may be present) are not representative of the expected molecular surface charge at infinite dilution. We found the exponential constant, *k*_*exp*_, derived from *η*_*rel*_*/c* exponential modeling, to correlate strongly with *k*_*D*_, aligning to the use of *k*_*D*_ in predicting viscosity in the low concentration regime.^5,8,9^ However, these correlations were reduced significantly when comparing to high concentration data, suggesting limits to predicting viscosity at dose-relevant concentrations. The biophysical behavior of each mAb is also formulation dependent and whilst the pH was within specification (pH 6 .0± 0.2) through tangential flow filtration steps, formulation composition was not determined and the Gibbs Donnan effect was not accounted for, which could impact protein charge.

Beyond size, pair interactions and charge, the shape factor (*R*_*g*_*/r*_*h*_) and computed shape coefficients were used to gauge morphological differences of the anti-IL-8 molecule panel (Supplementary Figure S7). No strong correlations were observed between these shape parameters, and *[η]* or high concentration viscosity. Both methods derived molecular volume information from homology constructs, which did not account for environmental differences (buffer components, surface counterions) nor the effect of neighboring molecules on molecular conformations.

### Limited predictive power of viscosity models

**Figure 5.**
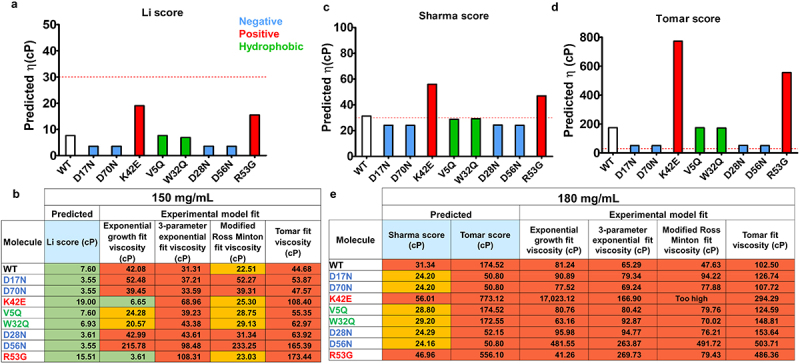
Predicted viscosity scores at 150 mg/mL Li (a) and Sharma (c) and Tomar (d) predicted viscosities at 180 mg/mL for anti-IL-8 wild-type (WT) and mutant IgG. Comparisons to experimental data at 150 mg/mL (**b**) and 180 mg/mL (**e**) with all model fits reported. Red dotted line represents an ‘acceptable’ viscosity threshold of 30 cP. Compared to wild-type, mutants targeting negative patches (blue bars) had consistently lower predicted viscosities for all models. Mutants targeting positive patches (red bars) had consistently higher predicted viscosities to WT, and hydrophobic patch disrupting mutants (green bars) had similar predicted viscosities to WT. For predicted versus experimental viscosity data, the following thresholds were set: ≤20 cP (green), 20cP≤≥30cP (amber) and ≥30cP (red).

The development of models derived from sequence- and structure- based molecular descriptors to predict and mitigate viscosity risks represents an ever-growing field of research. We iterated through testing simple regression models developed from mAb molecular descriptors of small datasets. Li^13^ and Sharma^14^ models underpredicted the viscosities of the anti-IL-8 molecule panel, whilst the Tomar^15^ model over-predicted (Figure 5), suggesting overfitting.

**Figure 6.**
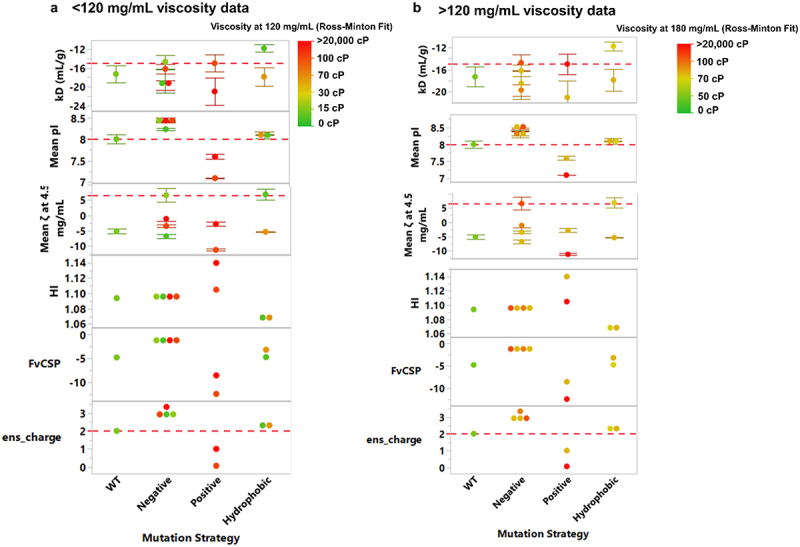
Experimental parameters and sequence-based molecular descriptors, categorized according to Kingsbury et al. ^45^ for anti-IL-8 IgGs, categorized by mutation strategy, across the high concentration (< 120 mg/mL) viscosity data (a) and ultra-high concentration (≥120 mg/mL) viscosity data (b). Ross-Minton model fitted experimental viscosities were used to grade each molecule from low viscosity to high viscosity (green to red) (Supplementary Table S7). Experimental parameters were the self-interaction parameter, *k*_*D*_, mean measured isoelectric point (pI) and mean zeta potential (ζ) at 5 mg/mL. Sequence-based molecular descriptors were hydrophobic index (HI) and fv charge symmetry (*F_v_*_*CSP*_). Ensemble charge is included as a structure-based descriptor that has previously been correlated with viscosity.^46^ red dotted lines represent thresholds attributed from clustering analysis from original studies where correlations with viscosity were found. Error bars represent standard deviation.

Furthermore, clustering based on the five descriptors identified by Kingsbury et al. ^45^ did not reveal consistent trends to categorize viscosity risks for the anti-IL-8 molecules at high or ultra-high concentrations (Figure 6). Classification using the ensemble charge parameter achieved accuracy rates ranging from 55% to 66% when predicting viscosity values at 120 mg/mL, depending on the concentration regime used for interpolation/extrapolation (Supplementary Table S8).

Generally, there is a consensus amongst these models that both electrostatic and hydrophobic parameters play a role in predicting viscosity, but the accuracy of these models relies heavily on the diversity and size of their datasets. As a result, machine learning approaches, leveraging comprehensive molecular descriptors and larger datasets often limited to clinical-phase mAbs for accessibility reasons, are gaining popularity. The Lai decision tree^58^ (Figure S8), incorporating a ‘high viscosity index’ (HVI) effectively classified the anti-IL-8 mutants as highly viscous, and the Makowski decision tree^18^ (Figure S9) classified negative-patch disrupting mutants (i.e. D→N) as low viscosity based on the predicted isoelectric point thresholds. Importantly, the prediction accuracy of these models varies depending on the fitting and interpolation methods for concentration-dependent viscosities, the concentration range examined, and the defined viscosity thresholds. While a consensus suggests a low viscosity threshold of 20–30 cP, some studies have proposed values as low as 15 cP.^60^ Confusion matrices evaluating the Lai and Makowski decision trees (Supplementary Table S9 and Table S10) illustrate how model choice, concentration range, and viscosity thresholds impact the proportion of true positives/negatives versus false positives/negatives.

**Figure 7.**
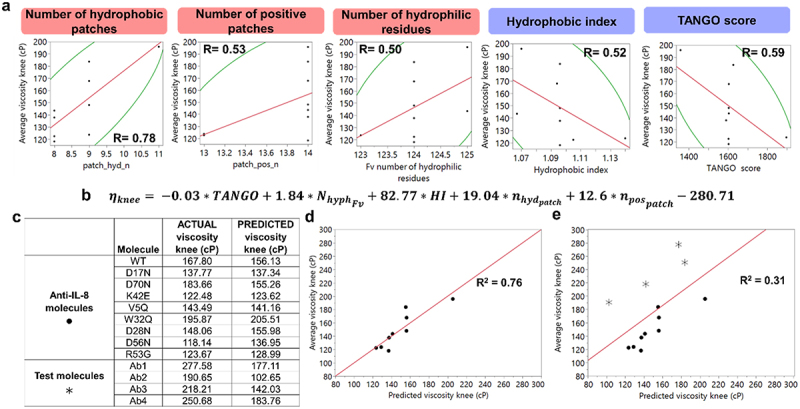
Viscosity regression model from an anti-IL-8 mAb panel lacked predictive ability with in-house test molecules. five molecular descriptor variables (a) were selected for modelling to predict the averaged knee for each viscosity profile per molecule. The respective scatter plots with Pearson correlation coefficients (R) per variable are reported. The resulting equation (b) was generated through non-linear iterative partial least square (NIPLS) regression with leave-one-out cross validation. Predicted viscosity knee values (c) were lower for the more viscous anti-IL-8 molecules compared to the less viscous test molecules. The model predicted the anti-IL-8 viscosity knee values well (R^2^=0.76, RMSE=12.7, *R*=0.87) (d), but underpredicted for the test set (R^2^=0.31, RMSE=43.8, *R*=0.56) (e).

We aimed to identify computational parameters predicting the knee of viscosity curves for the anti-IL-8 molecule panel. Strong correlations were found with the TANGO score, number of hydrophilic residues, hydrophobic index, and counts of hydrophobic and positive patches, encompassing electrostatic and hydrophobic profiling (Supplementary Figure S10). Using partial least-squares regression and leave-one-out cross validation (Figure 7), we developed a model demonstrated 0.76 R^2^ accuracy (RMSE = 12.7, Pearson’s *R* = 0.87), suggesting potential for defining key parameters for viscosity predictions in early-phase screening on a project-specific basis. However, when tested on a proprietary in-house molecule set, the model showed signs of overfitting to the mutant panel, echoing previous regression models’ limitations due to their focus on limited parameters. The model produced in Figure 7 provides an example of overfitting to small datasets of a panel of molecules with only small variations in their biophysical profiles from single-point mutations. This highlights the need for larger datasets incorporating diverse molecular scaffolds and mAbs with varying viscosities to build a robust predictive model.

## Conclusion

Knowledge of factors governing elevated solution viscosity at high mAb concentrations is critical in developing new mAbs for self-administration. Early measurements of solution viscosity are hindered by significant material-associated cost burdens. Therefore, a combination of predictive and experimental frameworks for solution viscosity prediction are required.

In this work, we address the question of which predictive and viscosity fitting models are optimal for viscosity prediction, using an anti-IL-8 IgG panel. We observed that the selection of fit models plays a critical role in the interpretation of viscosity results and the use of interpolated or extrapolated results carries significant variability risk. We found that extrapolation of viscosity measurements in a high concentration regime are not predictive of viscosities at ultra-high concentrations, suggesting various concentration-dependent mechanisms govern self-interaction, assembly, and aggregation. We highlighted the use of hydrodynamic and colloidal parameters can help elucidate what mechanisms drive viscosity in low concentration regimes, but these did not correlate to ultra-high concentration viscosities.

Predictive regression models from small datasets are generally overfitted and lack generalizability. We demonstrate the limitations of current machine learning models using global parameters which are insensitive to residue-level differences that impact viscosity. We propose the use of machine learning for viscosity predictions, incorporating amino acid sequences and structure-based descriptors and/or dilute solution data to improve the probability of identifying sequence motifs governing molecular properties which give rise to viscosity. We also recommend the use of ‘non-developable’ molecules in training and testing datasets to better account for biophysical risks in early-phase development.

## Supplementary Material

Supplementary_information_revised_4.docx

Supporting informationthird submission.xlsx

## References

[1] Jiskoot W, Hawe A, Menzen T, Volkin DB, Crommelin DJA. Ongoing challenges to develop high concentration monoclonal antibody-based formulations for subcutaneous administration: quo vadis? JPharmSci. 2022;111(4):861–17. doi:10.1016/j.xphs.2021.11.008. 34813800

[2] Yadav S, Shire SJ, Kalonia DS. Factors affecting the viscosity in high concentration solutions of different monoclonal antibodies. J Pharm Sci. 2010;99(12):4812–4829. doi:10.1002/jps.22190. 20821382

[3] Bhattad A. Review on viscosity measurement: devices, methods and models. J Therm Anal Calorim. 2023;148(14):6527–6543. doi:10.1007/s10973-023-12214-0.

[4] Prass TM, Garidel P, Blech M, Schäfer LV. Viscosity prediction of high-concentration antibody solutions with atomistic simulations. J Chem Inf Model. 2023;63(19):6129–6140. doi:10.1021/acs.jcim.3c00947. 37757589 PMC10565822

[5] Yadav S, Shire SJ, Kalonia DS. Viscosity behavior of high-concentration monoclonal antibody solutions: correlation with interaction parameter and electroviscous effects. J Pharm Sci. 2012;101(3):998–1011. doi:10.1002/jps.22831. 22113861

[6] Li J, Cheng Y, Chen X, Zheng S. Impact of electroviscous effect on viscosity in developing highly concentrated protein formulations: lessons from non-protein charged colloids. Int J Pharm X. 2019;1:100002. doi:10.1016/j.ijpx.2018.100002. 31545855 PMC6733305

[7] Arzenšek D, Kuzman D, Podgornik R. Colloidal interactions between monoclonal antibodies in aqueous solutions. J Colloid Interface Sci. 2012;384(1):207–216. doi:10.1016/j.jcis.2012.06.055. 22840854

[8] Saito S, Hasegawa J, Kobayashi N, Kishi N, Uchiyama S, Fukui K. Behavior of monoclonal antibodies: relation between the second virial coefficient (B 2) at low concentrations and aggregation propensity and viscosity at high concentrations. Pharm Res. 2012;29(2):397–410. doi:10.1007/s11095-011-0563-x. 21853361

[9] Tomar DS, Kumar S, Kumar S, Singh S, Singh SK. Molecular basis of high viscosity in concentrated antibody solutions: strategies for high concentration drug product development. mAbs. 2016;8(2):216–228. doi:10.1080/19420862.2015.1128606. 26736022 PMC5074600

[10] Chowdhury A, Bollinger JA, Dear BJ, Cheung JK, Johnston KP, Truskett TM. Coarse-grained molecular dynamics simulations for understanding the impact of short-range anisotropic attractions on structure and viscosity of concentrated monoclonal antibody solutions. Mol Pharm. 2020;17(5):1748–1756. doi:10.1021/acs.molpharmaceut.9b00960. 32101441

[11] Lai P-K, Swan JW, Trout BL. Calculation of therapeutic antibody viscosity with coarse-grained models, hydrodynamic calculations and machine learning-based parameters. mAbs. 2021;13(1):1907882. doi:10.1080/19420862.2021.1907882. 33834944 PMC8043186

[12] Skar-Gislinge N, Camerin F, Stradner A, Zaccarelli E, Schurtenberger P. Using cluster theory to calculate the experimental structure factors of antibody solutions. Mol Pharm. 2023;20(5):2738–2753. doi:10.1021/acs.molpharmaceut.3c00191. 37067466 PMC10155212

[13] Li L, Kumar S, Buck PM, Burns C, Lavoie J, Singh SK, Warne NW, Nichols P, Luksha N, Boardman D. Concentration dependent viscosity of monoclonal antibody solutions: explaining experimental behavior in terms of molecular properties. Pharm Res. 2014;31(11):3161–3178. doi:10.1007/s11095-014-1409-0. 24906598

[14] Sharma VK, Patapoff TW, Kabakoff B, Pai S, Hilario EC, Zhang B, Li C, Borisov O, Kelley RF, Chorny I, et al. In silico selection of therapeutic antibodies for development: viscosity, clearance, and chemical stability. Proc Natl Acad Sci USA. 2014;111(52):18601–18606. doi:10.1073/pnas.1421779112. 25512516 PMC4284567

[15] Tomar DS, Li L, Broulidakis MP, Luksha NG, Burns CT, Singh SK, Kumar S. In-silico prediction of concentration-dependent viscosity curves for monoclonal antibody solutions. mAbs. 2017;9(3):476–489. doi:10.1080/19420862.2017.1285479. 28125318 PMC5384706

[16] Schmitt J, Razvi A, Grapentin C. Predictive modeling of concentration-dependent viscosity behavior of monoclonal antibody solutions using artificial neural networks. mAbs. 2023;15(1):2169440. doi:10.1080/19420862.2023.2169440. 36705325 PMC9888472

[17] Lai P-K. DeepSCM: an efficient convolutional neural network surrogate model for the screening of therapeutic antibody viscosity. Comput Struct Biotechnol J. 2022;20:2143–2152. doi:10.1016/j.csbj.2022.04.035. 35832619 PMC9092385

[18] Makowski EK, Chen H-T, Wang T, Wu L, Huang J, Mock M, Underhill P, Pelegri-O’Day E, Maglalang E, Winters D, et al. Reduction of monoclonal antibody viscosity using interpretable machine learning. mAbs. 2024;16(1):2303781. doi:10.1080/19420862.2024.2303781. 38475982 PMC10939158

[19] Armstrong GB, Shah V, Sanches P, Patel M, Casey R, Jamieson C, Burley GA, Lewis W, Rattray Z. A framework for the biophysical screening of antibody mutations targeting solvent-accessible hydrophobic and electrostatic patches for enhanced viscosity profiles. Comput Struct Biotechnol J. 2024;23:2345–2357. doi:10.1016/j.csbj.2024.05.041. 38867721 PMC11167247

[20] Fernandez-Escamilla A-M, Rousseau F, Schymkowitz J, Serrano L. Prediction of sequence-dependent and mutational effects on the aggregation of peptides and proteins. Nat Biotechnol. 2004;22(10):1302–1306. doi:10.1038/nbt1012. 15361882

[21] Linding R, Schymkowitz J, Rousseau F, Diella F, Serrano L. A comparative study of the relationship between protein structure and β-aggregation in globular and intrinsically disordered proteins. J Mol Biol. 2004;342(1):345–353. doi:10.1016/j.jmb.2004.06.088. 15313629

[22] Maurer-Stroh S, Debulpaep M, Kuemmerer N, Lopez de la Paz M, Martins IC, Reumers J, Morris KL, Copland A, Serpell L, Serrano L, et al. Exploring the sequence determinants of amyloid structure using position-specific scoring matrices. Nat Methods. 2010;7(3):237–242. doi:10.1038/nmeth.1432. 20154676

[23] Oliveberg M. Waltz, an exciting new move in amyloid prediction. Nat Methods. 2010;7(3):187–188. doi:10.1038/nmeth0310-187. 20195250

[24] Agrawal NJ, Helk B, Kumar S, Mody N, Sathish HA, Samra HS, Buck PM, Li L, Trout BL. Computational tool for the early screening of monoclonal antibodies for their viscosities. mAbs. 2016;8(1):43–48. doi:10.1080/19420862.2015.1099773. 26399600 PMC4966561

[25] Eisenberg D, Schwarz E, Komaromy M, Wall R. Analysis of membrane and surface protein sequences with the hydrophobic moment plot. J Mol Biol. 1984;179(1):125–142. doi:10.1016/0022-2836(84)90309-7. 6502707

[26] Connolly BD, Petry C, Yadav S, Demeule B, Ciaccio N, Moore JMR, Shire SJ, Gokarn YR. Weak interactions govern the viscosity of concentrated antibody solutions: high-throughput analysis using the diffusion interaction parameter. Biophys J. 2012;103(1):69–78. doi:10.1016/j.bpj.2012.04.047. 22828333 PMC3388210

[27] Lai P-K, Gallegos A, Mody N, Sathish HA, Trout BL. Machine learning prediction of antibody aggregation and viscosity for high concentration formulation development of protein therapeutics. mAbs. 2022;14(1):2026208. doi:10.1080/19420862.2022.2026208. 35075980 PMC8794240

[28] Schwenger W, Pellet C, Attonaty D, Authelin J-R. An empirical quantitative model describing simultaneously temperature and concentration effects on protein solution viscosity. JPharmSci. 2020;109(3):1281–1287. doi:10.1016/j.xphs.2019.12.001. 31821824

[29] Ross PD, Minton AP. Hard quasispherical model for the viscosity of hemoglobin solutions. Biochem Biophys Res Commun. 1977;76(4):971–976. doi:10.1016/0006-291X(77)90950-0. 20088

[30] Apgar JR, Tam ASP, Sorm R, Moesta S, King AC, Yang H, Kelleher K, Murphy D, D’Antona AM, Yan G, et al. Modeling and mitigation of high-concentration antibody viscosity through structure-based computer-aided protein design. PLOS ONE. 2020;15(5):e0232713. doi:10.1371/journal.pone.0232713. 32379792 PMC7205207

[31] Kanai S, Liu J, Patapoff TW, Shire SJ. Reversible self-association of a concentrated monoclonal antibody solution mediated by fab–fab interaction that impacts solution viscosity. J Pharm Sci. 2008;97(10):4219–4227. doi:10.1002/jps.21322. 18240303

[32] Wang W, Lilyestrom WG, Hu ZY, Scherer TM. Cluster size and quinary structure determine the rheological effects of antibody self-association at high concentrations. J Phys Chem B. 2018;122(7):2138–2154. doi:10.1021/acs.jpcb.7b10728. 29359938

[33] Roche A, Gentiluomo L, Sibanda N, Roessner D, Friess W, Trainoff SP, Curtis R. Towards an improved prediction of concentrated antibody solution viscosity using the Huggins coefficient. J Colloid Interface Sci. 2022;607:1813–1824. doi:10.1016/j.jcis.2021.08.191. 34624723

[34] García De La Torre J, Hernández Cifre JG. Hydrodynamic properties of Biomacromolecules and macromolecular complexes: concepts and methods. A tutorial mini-review. J Mol Biol. 2020;432(9):2930–2948. doi:10.1016/j.jmb.2019.12.027. 31877325

[35] Ortega A, Amorós D, García de la Torre J. Prediction of hydrodynamic and other solution properties of rigid proteins from atomic- and residue-level models. Biophys J. 2011;101(4):892–898. doi:10.1016/j.bpj.2011.06.046. 21843480 PMC3175065

[36] Tian Z, Jiang X, Chen Z, Huang C, Qian F. Quantifying protein shape to elucidate its influence on solution viscosity in high-concentration electrolyte solutions. Mol Pharm. 2024;21(4):1719–1728. doi:10.1021/acs.molpharmaceut.3c01075. 38411904

[37] Lilyestrom WG, Yadav S, Shire SJ, Scherer TM. Monoclonal antibody self-association, cluster formation, and rheology at high concentrations. J Phys Chem B. 2013;117(21):6373–6384. doi:10.1021/jp4008152. 23560896

[38] Roche A. Intermolecular interactions and rheological properties in monoclonal antibody solutions [thesis]. University of Manchester; 2021.

[39] García De La Torre J, Huertas ML, Carrasco B. Calculation of hydrodynamic properties of globular proteins from their atomic-level structure. Biophys J. 2000;78(2):719–730. doi:10.1016/S0006-3495(00)76630-6. 10653785 PMC1300675

[40] Abdelmohsen LKEA, Rikken RSM, Christianen PCM, Van Hest JCM, Wilson DA. Shape characterization of polymersome morphologies via light scattering techniques. Polymer. 2016;107:445–449. doi:10.1016/j.polymer.2016.06.067.

[41] Muza UL, Williams CD, Lederer A. Unravelling the thermo-responsive evolution from single-chain to multiple-chain nanoparticles by thermal field-flow fractionation. Polym Chem. 2023;14(28):3302–3308. doi:10.1039/D3PY00426K.

[42] Hydrodynamic Radius - Radius of Gyration | Malvern Panalytical. [accessed 2024 06 11]. https://www.malvernpanalytical.com/en/learn/knowledge-center/insights/size-matters-rh-versus-rg.

[43] Meng Kok C, Rudin A. Relationship between the hydrodynamic radius and the radius of gyration of a polymer in solution. Makromol Chem Rapid Commun. 1981;2(11):655–659. doi:10.1002/marc.1981.030021102.

[44] Kingsbury JS, Saini A, Auclair SM, Fu L, Lantz MM, Halloran KT, Calero-Rubio C, Schwenger W, Airiau CY, Zhang J, et al. A single molecular descriptor to predict solution behavior of therapeutic antibodies. Sci Adv. 2020;6(32):eabb0372. doi:10.1126/sciadv.abb0372. 32923611 PMC7457339

[45] Thorsteinson N, Gunn JR, Kelly K, Long W, Labute P. Structure-based charge calculations for predicting isoelectric point, viscosity, clearance, and profiling antibody therapeutics. MAbs. 2021;13(1):1981805. doi:10.1080/19420862.2021.1981805. 34632944 PMC8510563

[46] Chow C-K, Allan BW, Chai Q, Atwell S, Lu J. Therapeutic antibody engineering to improve viscosity and phase separation guided by crystal structure. Mol Pharm. 2016;13(3):915–923. doi:10.1021/acs.molpharmaceut.5b00817. 26849155

[47] Tilegenova C, Izadi S, Yin J, Huang CS, Wu J, Ellerman D, Hymowitz SG, Walters B, Salisbury C, Carter PJ. Dissecting the molecular basis of high viscosity of monospecific and bispecific IgG antibodies. mAbs. 2020;12(1):1692764. doi:10.1080/19420862.2019.1692764. 31779513 PMC6927759

[48] Wang S, Zhang N, Hu T, Dai W, Feng X, Zhang X, Qian F. Viscosity-lowering effect of amino acids and salts on highly concentrated solutions of two IgG1 monoclonal antibodies. Mol Pharm. 2015;12(12):4478–4487. doi:10.1021/acs.molpharmaceut.5b00643. 26528726

[49] Hong T, Iwashita K, Shiraki K. Viscosity control of protein solution by small solutes: a review. CPPS. 2018;19(8):746–758. doi:10.2174/1389203719666171213114919. PMC6182935 29237380

[50] Rodrigues D, Tanenbaum LM, Thirumangalathu R, Somani S, Zhang K, Kumar V, Amin K, Thakkar SV. Product-specific impact of viscosity modulating formulation excipients during ultra-high concentration biotherapeutics drug product development. J Pharm Sci. 2021;110(3):1077–1082. doi:10.1016/j.xphs.2020.12.016. 33340533

[51] Yadav S, Laue TM, Kalonia DS, Singh SN, Shire SJ. The influence of charge distribution on self-association and viscosity behavior of monoclonal antibody solutions. Mol Pharm. 2012;9(4):791–802. doi:10.1021/mp200566k. 22352470

[52] Stradner A, Schurtenberger P. Potential and limits of a colloid approach to protein solutions. Soft Matter. 2020;16(2):307–323. doi:10.1039/C9SM01953G. 31830196

[53] Garidel P, Blume A, Wagner M. Prediction of colloidal stability of high concentration protein formulations. Pharm Devel Technol. 2015;20(3):367–374. doi:10.3109/10837450.2013.871032. 24392929

[54] Sudrik C, Cloutier T, Pham P, Samra HS, Trout BL. Preferential interactions of trehalose, L-Arginine.HCl and sodium chloride with therapeutically relevant IgG1 monoclonal antibodies. mAbs. 2017;9(7):1155–1168. doi:10.1080/19420862.2017.1358328. 28758834 PMC5627590

[55] Yearley EJ, Godfrin PD, Perevozchikova T, Zhang H, Falus P, Porcar L, Nagao M, Curtis JE, Gawande P, Taing R, et al. Observation of small cluster formation in concentrated monoclonal antibody solutions and its implications to solution viscosity. Biophys J. 2014;106(8):1763–1770. doi:10.1016/j.bpj.2014.02.036. 24739175 PMC4008822

[56] Stone-Masui J, Watillon A. Electroviscous effects in dispersions of monodisperse polystyrene latices. J Colloid Interface Sci. 1968;28(2):187–202. doi:10.1016/0021-9797(68)90120-3.

[57] Lai P-K, Fernando A, Cloutier TK, Gokarn Y, Zhang J, Schwenger W, Chari R, Calero-Rubio C, Trout BL. Machine learning applied to determine the molecular descriptors responsible for the viscosity behavior of concentrated therapeutic antibodies. Mol Pharm. 2021;18(3):1167–1175. doi:10.1021/acs.molpharmaceut.0c01073. 33450157

[58] Mock M, Jacobitz AW, Langmead CJ, Sudom A, Yoo D, Humphreys SC, Alday M, Alekseychyk L, Angell N, Bi V, et al. Development of in silico models to predict viscosity and mouse clearance using a comprehensive analytical data set collected on 83 scaffold-consistent monoclonal antibodies. mAbs. 2023;15(1):2256745. doi:10.1080/19420862.2023.2256745. 37698932 PMC10498806

