# Supplementary material for: Reconciling predicted and measured viscosity parameters in high concentration therapeutic antibody solutions
Source: MAbs. 2024 Dec 11;16(1):2438172. doi: 10.1080/19420862.2024.2438172 (PMC11790245; doi:10.1080/19420862.2024.2438172)
Supplement: Supplementary_information_revised_4.docx [file KMAB_A_2438172_SM9818.docx]

**Supplementary Information for Reconciling predicted and measured viscosity parameters in high concentration antibody solutions**

Georgina Bethany Armstrong,^1,3*^ Aisling Roche^2^, William Lewis^1^, Zahra Rattray^3*^

1. *Drug Substance Development, GlaxoSmithKline, Gunnels Wood Road, Stevenage, UK.*
2. *Large Molecule Discovery, GlaxoSmithKline, Gunnel Woods Road, Stevenage, UK*
3. *Strathclyde Institute of Pharmacy and Biomedical Sciences, University of Strathclyde, Glasgow, UK.*

*corresponding author(s): Georgina Armstrong ([Georgina.x.armstrong@gsk.com](mailto:Georgina.x.armstrong@gsk.com)), Zahra Rattray ([Zahra.rattray@strath.ac.uk](mailto:Zahra.rattray@strath.ac.uk)).

**Table S1 In silico molecular descriptors for anti-IL8 molecules computed from Fv homology constructs.**

| **Molecule** | **net_charge** | **Predicted ζ at Deybe length (mV)** | **ens_charge** | **pI_seq** | **pI_3D** | **VL net charge** | **VH net charge** | **FvCSP** | **Hydrophobic index** | **TANGO score** | **WALTZ score** | **Deep SCM** |
| --- | --- | --- | --- | --- | --- | --- | --- | --- | --- | --- | --- | --- |
| **WT** | 0.05 | 0.19 | 2.01 | 6.42 | 6.23 | -1.23 | 3.93 | -4.83 | 1.094 | 1603.94 | 486.574 | 1197.42 |
| **D17N (FWRL)** | 0.62 | 1.79 | 3.30 | 6.68 | 7.61 | -0.32 | 3.93 | -1.26 | 1.096 | 1590.12 | 486.574 | 1164.43 |
| **D70N (FWRL)** | 0.63 | 1.58 | 2.90 | 6.68 | 7.61 | -0.32 | 3.93 | -1.26 | 1.096 | 1627.07 | 486.574 | 1136.54 |
| **K42E (FWRL)** | -1.81 | -4.14 | 0.07 | 5.62 | 4.56 | -3.16 | 3.93 | -12.42 | 1.105 | 1602.38 | 486.574 | 1253.89 |
| **V5Q (FWRH)** | 0.05 | 0.19 | 2.25 | 6.42 | 6.23 | -1.23 | 3.83 | -4.71 | 1.067 | 1603.94 | 486.574 | 1194.19 |
| **W32Q (CDRH)** | 0.05 | 0.21 | 2.38 | 6.42 | 6.24 | -1.22 | 3.92 | -4.78 | 1.070 | 1357.71 | 389.25 | 1213.87 |
| **D28N (CDRH)** | 0.62 | 1.46 | 2.88 | 6.68 | 7.58 | -0.34 | 3.93 | -1.34 | 1.096 | 1603.43 | 486.574 | 1062.51 |
| **D56N (CDRH)** | 0.63 | 1.51 | 3.00 | 6.68 | 7.61 | -0.32 | 3.82 | -1.22 | 1.096 | 1602.95 | 486.574 | 1136.34 |
| **R53G (CDRH)** | -0.83 | -1.96 | 1.01 | 6.07 | 4.93 | -2.18 | 3.93 | -8.57 | 1.140 | 1897.13 | 486.574 | 1256.43 |

**Table S2 Parameters derived from four viscosity model fits applied to concentration-viscosity profiles for mutant variants and WT molecule.**

| **Molecule** | **Concentration regime (mg/mL)** | **Exponential growth** | | | **3-parameter exponential** | | | **Modified Ross Minton** | | **Tomar** | | |
| --- | --- | --- | --- | --- | --- | --- | --- | --- | --- | --- | --- | --- |
|  |  | **Y0** | **k** | **Tau (1/k)** | **Slope A3 (K^-1^)** | **Slope A2** | **A1 (cP)** | **k/v** | **[η] mL/g** | **η_0_** | **B** | **LnA** |
| **WT** | <120 | 0.848 | 0.022 | 44.675 | 0.000 | 0.021 | -0.02879 | 0.190 | 14.131 | 1.13 | 0.020822 | -0.15101 |
|  | >120 | 1.568 | 0.022 | 45.598 | 0.000 | 0.024 | -0.23068 | 0.068 | 16.588 | 1.13 | 0.024497 | -0.3529 |
| **D17N** | <120 | 0.031 | 0.063 | 15.947 | 0.000 | 0.026 | -0.17547 | 1.086 | 6.785 | 1.13 | 0.025749 | -0.29769 |
|  | >120 | 3.368 | 0.018 | 54.621 | 0.000 | 0.025 | -0.16936 | -0.052 | 31.986 | 1.13 | 0.025239 | -0.29158 |
| **D70N** | <120 | 1.145 | 0.015 | 64.995 | 0.000 | 0.015 | 0.178279 | 0.025 | 14.824 | 1.13 | 0.014684 | 0.056062 |
|  | >120 | 1.347 | 0.023 | 44.412 | 0.000 | 0.024 | -0.10261 | -0.009 | 24.418 | 1.13 | 0.024112 | -0.22483 |
| **K42E** | <120 | 0.027 | 0.066 | 15.062 | 0.000 | 0.026 | 0.04139 | 0.666 | 9.623 | 1.13 | 0.026442 | -0.08083 |
|  | >120 | 0.000 | 0.262 | 3.823 | 0.000 | 0.029 | -0.18578 | 1.247 | 4.251 | 1.13 | 0.029462 | -0.308 |
| **V5Q** | <120 | 0.779 | 0.032 | 31.297 | 0.000 | 0.026 | 0.072141 | 0.297 | 15.342 | 1.13 | 0.026131 | -0.05008 |
|  | >120 | 0.06 | 0.040 | 24.959) | 0.000 | 0.024 | 0.079578 | 0.135 | 15.003 | 1.13 | 0.023932 | -0.04264 |
| **W32Q** | <120 | 1.494 | 0.012 | 83.143 | 0.000 | 0.015 | 0.276493 | 0.000 | 17.090 | 1.13 | 0.015001 | 0.154276 |
|  | >120 | 0.075 | 0.037 | 26.748 | 0.000 | 0.025 | -0.03551 | 0.085 | 16.992 | 1.13 | 0.025371 | -0.15773 |
| **D28N** | <120 | 0.797 | 0.026 | 38.135 | 0.000 | 0.022 | 0.011487 | 0.696 | 9.420 | 1.13 | 0.021863 | -0.11073 |
|  | >120 | 0.775 | 0.027 | 37.353 | 0.000 | 0.026 | -0.1051 | 0.080 | 17.500 | 1.13 | 0.02587 | -0.22732 |
| **D56N** | <120 | 0.852 | 0.024 | 41.947 | 0.000 | 0.016 | 0.248634 | 0.283 | 12.832 | 1.13 | 0.016293 | 0.126416 |
|  | >120 | 3.898 | 0.027 | 37.371 | 0.000 | 0.033 | -0.33795 | -0.049 | 48.246 | 1.13 | 0.032852 | -0.46016 |
| **R53G** | <120 | 1.031 | 0.029 | 33.907 | 0.000 | 0.025 | 0.287381 | 18.269 | 0.577 | 1.13 | 0.024674 | 0.165163 |
|  | >120 | 0.000 | 0.081 | 12.317 | 0.000 | 0.030 | 0.122623 | 0.248 | 11.507 | 1.13 | 0.030416 | 0.000405 |

**Table S3 Molecule rankings in order of increasing viscosity at 180 mg/mL extrapolated or interpolated per concentration regime and per model equation fit.**

| **Growth exponential** | | **3-parameter exponential** | | **Modified Ross-Minton** | | **Tomar** |  |
| --- | --- | --- | --- | --- | --- | --- | --- |
| **High concentration** | **Ultra-high concentration viscosity** | **High concentration** | **Ultra-high concentration viscosity** | **High concentration** | **Ultra-high concentration viscosity** | **High concentration** | **Ultra-high concentration viscosity** |
| W32Q (CDRH) | R53G (CDRL) | D70N (FWRL) | WT | D70N (FWRL) | WT | D70N (FWRL) | WT |
| D70N (FWRL) | W32Q (CDRH) | W32Q (CDRH) | D70N (FWRL) | W32Q (CDRH) | W32Q (CDRH) | W32Q (CDRH) | D70N (FWRL) |
| WT | D70N (FWRL) | D56N (CDRL) | D17N (FWRL) | WT | D28N (CDRL) | D56N (CDRL) | V5Q (FWRH) |
| D56N (CDRL) | V5Q (FWRH) | WT | V5Q (FWRH) | D56N (CDRL) | D70N (FWRL) | WT | D17N (FWRL) |
| D28N (CDRL) | WT | V5Q (FWRH) | W32Q (CDRH) | D17N (FWRL) | R53G (CDRL) | D28N (CDRL) | W32Q (CDRH) |
| R53G (CDRL) | D17N (FWRL) | D28N (CDRL) | D28N (CDRL) | V5Q (FWRH) | V5Q (FWRH) | D17N (FWRL) | D28N (CDRL) |
| V5Q (FWRH) | D28N (CDRL) | D17N (FWRL) | K42E (FWRL) | D28N (CDRL) | D17N (FWRL) | R53G (CDRL) | K42E (FWRL) |
| D17N (FWRL) | D56N (CDRL) | R53G (CDRL) | D56N (CDRL) | K42E (FWRL) | D56N (CDRL) | V5Q (FWRH) | R53G (CDRL) |
| K42E (FWRL) | K42E (FWRL) | K42E (FWRL) | R53G (CDRL) | R53G (CDRL) | K42E (FWRL) | K42E (FWRL) | D56N (CDRL) |

**Determination of the Huggins and Kraemer Parameters**


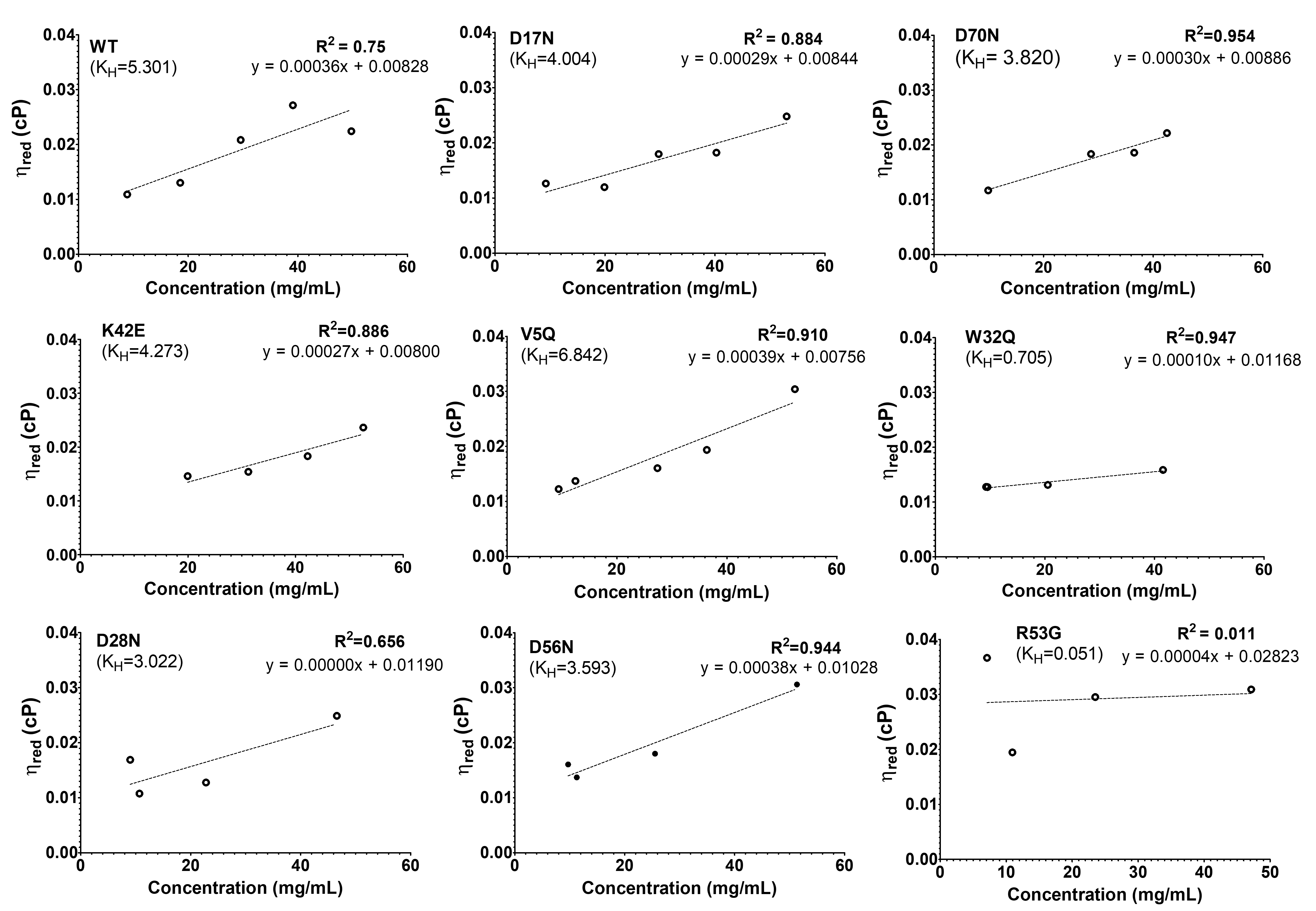


**Figure S1** Individual linear regression plots of reduced viscosity (η_sp_/c) versus concentration profiles used to derive the Huggins coefficient for the anti-IL8 panel. The Huggins coefficient (K_H_) was determined from the intrinsic viscosity (intercept) and slope of the plot. R^2^ values are reported to show goodness of fit to linear regression model.


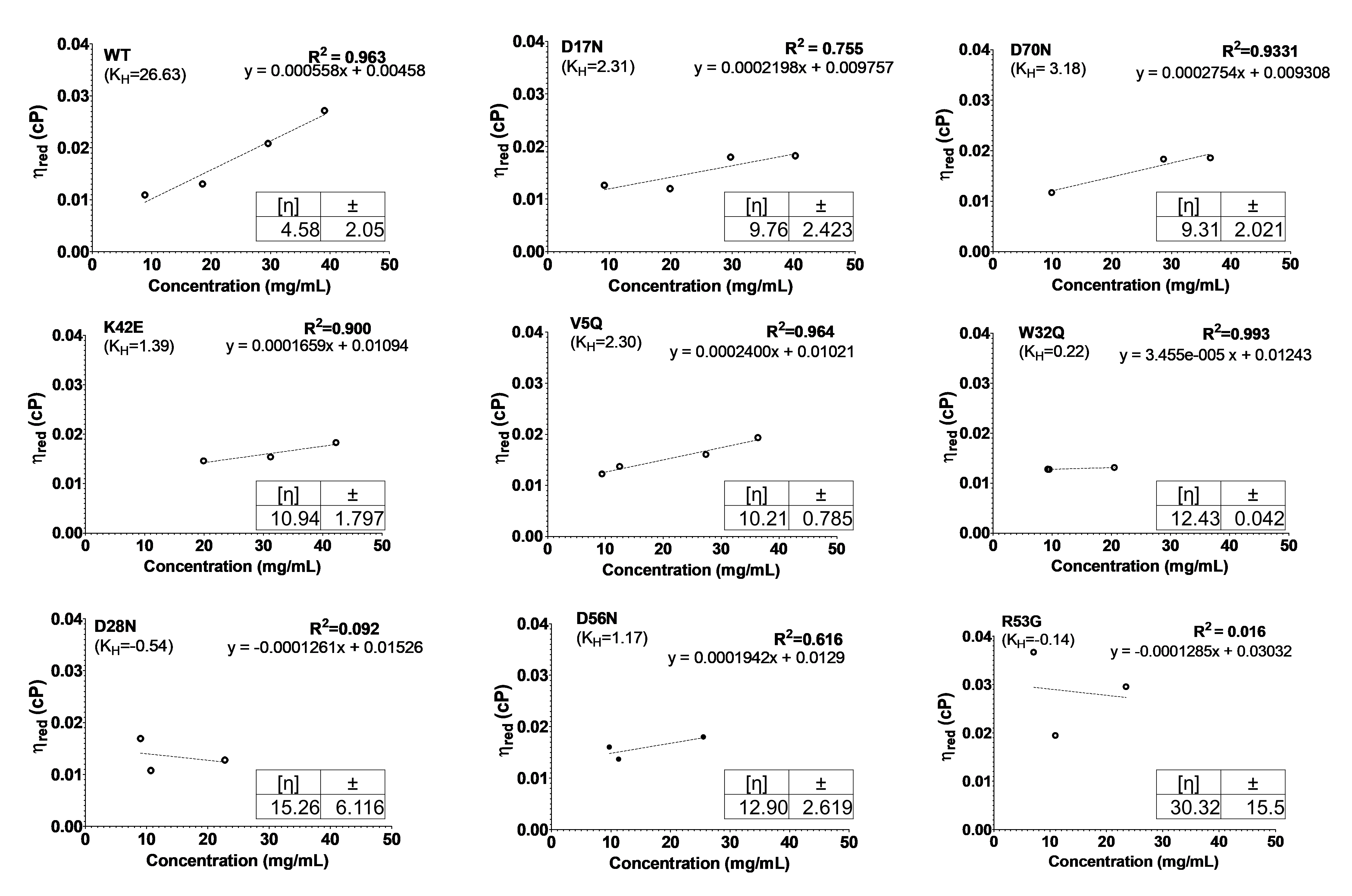


**Figure S2 Reduced viscosity (η_red_) (cP) over concentration (mg/mL) plots for anti-IL8 mutants with highest concentration data points removed.** Intrinsic viscosities ([η]_avg_) were derived from the intercept of the linear regression and used with the slope to derive Huggins coefficients (K_H_). R^2^ goodness fits to the linear regression model are shown.


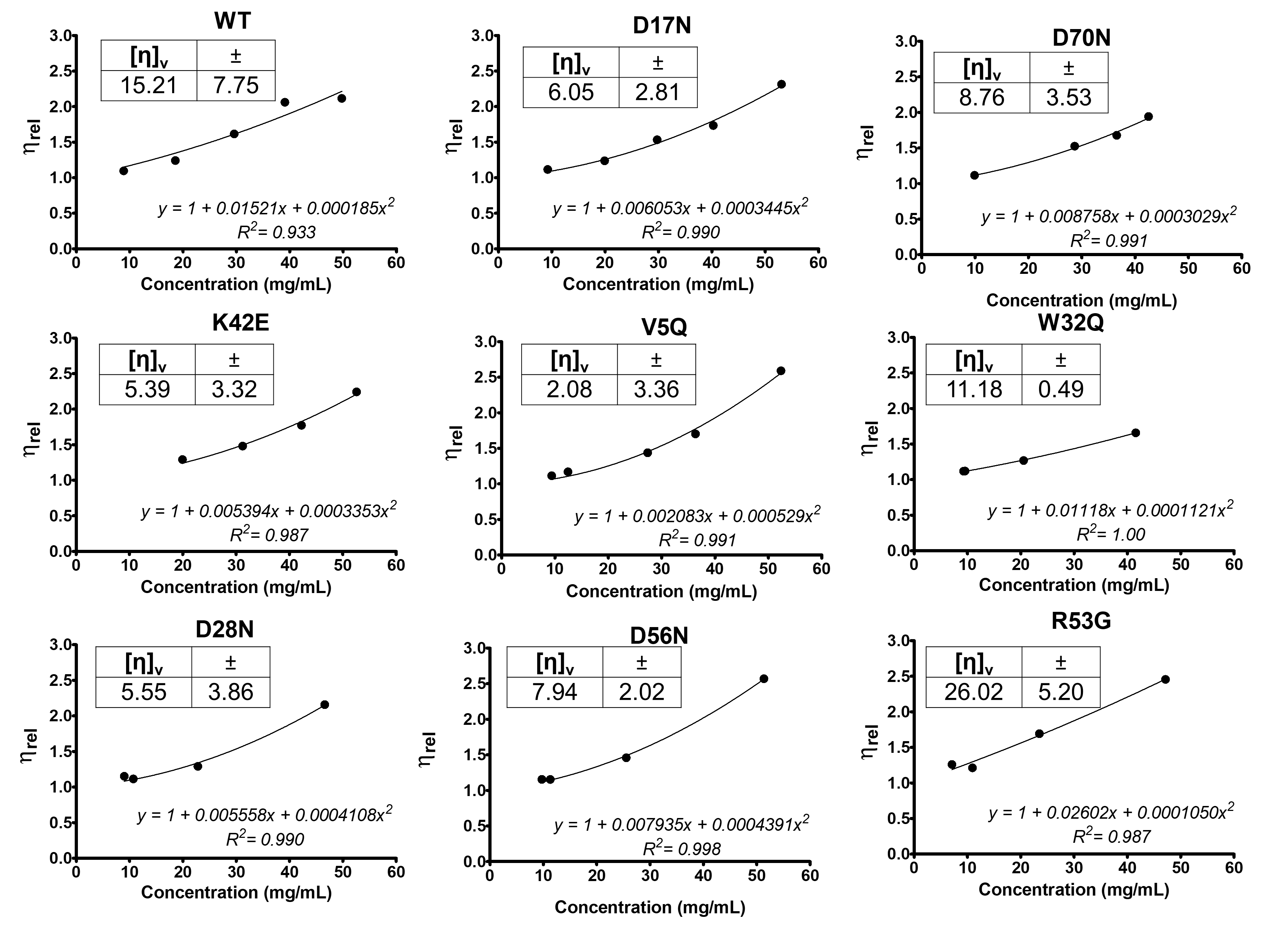


**Figure S3 Individual second order polynomial (quadratic) plots of relative viscosity versus concentration profiles used to derive [η]_V_ for the anti-IL8 mutant panel.**  K1 was constrained to 1 so [η]_v_ could not be negative.

**Figure S4 Correlation between intrinsic viscosities derived from linear regression ([n]_avg_) and polynomial non-linear fitting ([n]_v_).** Error bars represent mean standard error of intercepts from each respective model fit. Goodness of fit R^2^ reported.

**Table S4** **Mean intrinsic viscosity, [η]_avg_, k_exp_, and Huggins coefficients determined for anti-IL-8 mAbs.** Linear regression of the reduced viscosity-concentration was performed to determine the Huggins intrinsic viscosity, [η]_H_, and Huggins coefficient, k_H_. Second-order polynomial fitting of relative viscosities was also used to obtain alternate intrinsic viscosities ([n]_v_). The exponential constant, k_exp_, is shown from fitting relative viscosity (ηrel) data. Molecular weights are from mass spectrometry peptide mapping experiments and were used in the HYDROPRO program for [η] and radius of gyration (Rg) predictions. Values are represented as mean (± standard deviation), N=4.

|  |  |  |  |  |  | ***HYDROPRO Residue-shell mode*** | | ***HYDROPRO Residue-bead mode*** | |
| --- | --- | --- | --- | --- | --- | --- | --- | --- | --- |
| **Molecule** | **[η]_avg_ (mL/g)** | **[η]_v_ (mL/g)** | **k_exp_** | **Huggins coefficient, k_H_** | **Molecular weight (Da)** | **[η] (mL/g)** | **R_g_ (nm)** | **[η] (mL/g)** | **R_g_ (nm)** |
| WT | 8.28 (±3.89) | 15.21 (±7.75) | 15.78 | 5.30(±1.77) | 145276 | 7.426 | 5.32 | 7.165 | 5.102 |
| D17N (FWL) | 8.44 (±2.03) | 6.05 (±2.81) | 14.39 | 4.00(±0.53) | 145274 | 7.426 | 5.253 | 7.09 | 5.045 |
| D70N (FWL) | 8.86 (±1.49) | 8.76 (±3.53) | 14.71 | 3.82(±0.80) | 145274 | 7.43 | 5.279 | 7.079 | 5.057 |
| K42E (FWL) | 8.00 (±2.67) | 5.39 (±3.32) | 14.09 | 4.27(±1.27) | 145278 | 7.424 | 5.259 | 7.144 | 5.092 |
| V5Q (FWH) | 7.56 (±2.26) | 2.08 (±3.36) | 15.95 | 6.84(±2.1) | 145334 | 7.281 | 5.27 | 7.047 | 5.069 |
| W32Q (CDRH2) | 11.68 (±0.39) | 11.18 (±0.49) | 12.04 | 0.71(±0.11) | 145160 | 7.436 | 5.259 | 7.097 | 5.062 |
| D28N (CDRL1) | 9.83 (±4.02) | 5.55 (±3.86) | 14.86 | 3.02(±0.54) | 145274 | 7.44 | 5.272 | 7.111 | 5.102 |
| D56N (CDRL2) | 10.28 (±1.95) | 7.94 (±2.02) | 17.06 | 3.59(±0.81) | 145274 | 7.361 | 5.243 | 7.062 | 5.068 |
| R53G (CDRL1) | 28.23 (±7.56) | 26.02 (±5.20) | 20.32 | 0.05 (±0.09) | 145078 | 7.571 | 5.3 | 7.174 | 5.108 |


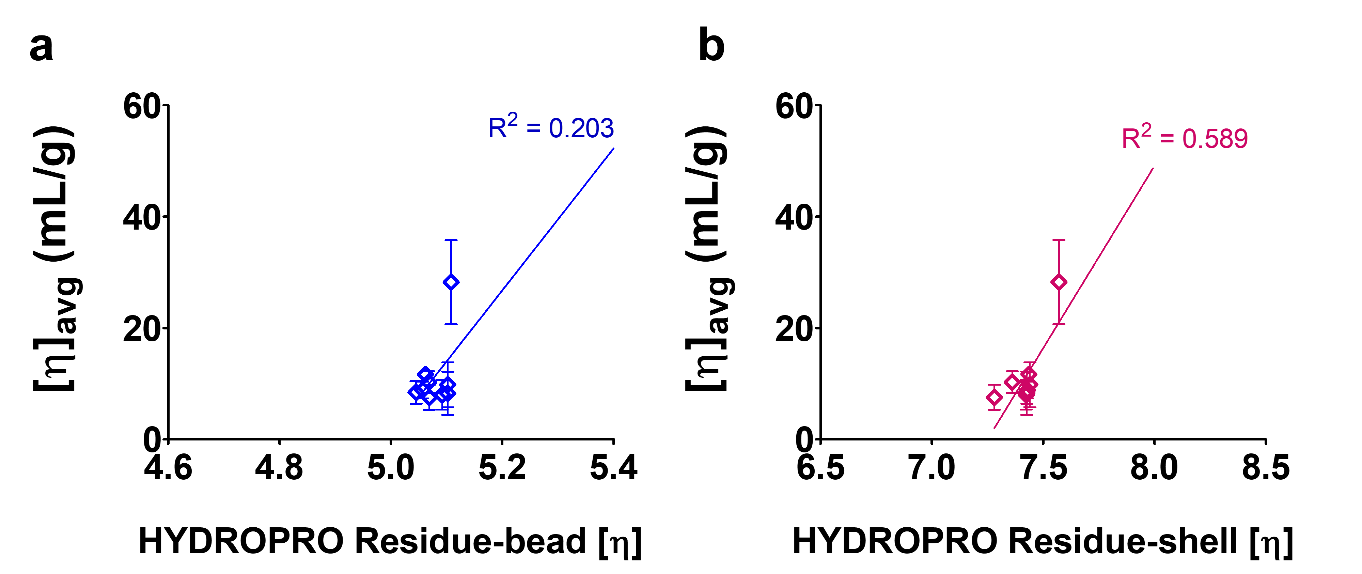


**Figure S5 Correlations of intrinsic viscosity from HYDROPRO program to experimental intrinsic viscosity ([η]_avg_).** Both residue-level modes were selected; residue-bead **(a)** and residue-shell **(b)**, and the residue-shell predictions had a stronger correlation to [η]_avg_. Linear goodness of fit R^2^ reported. Error bars represent standard error of [η]_avg_.


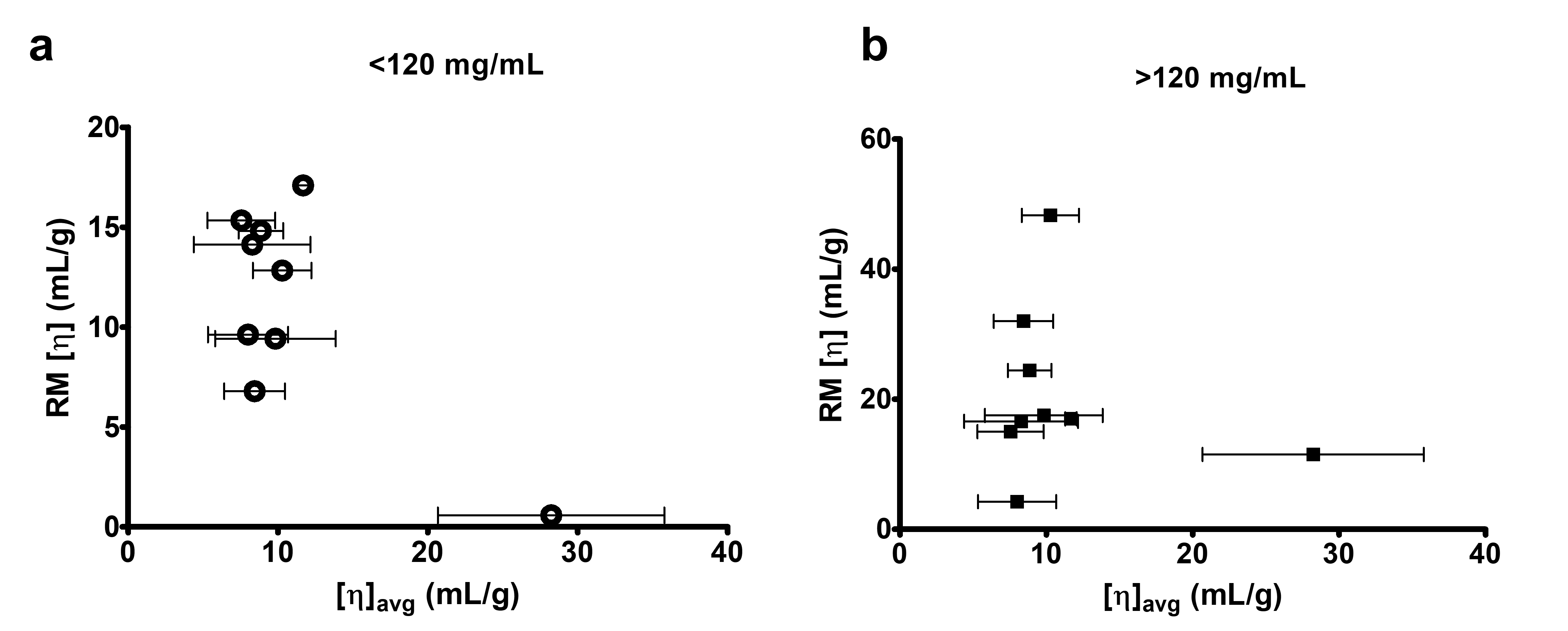


**Figure S6 Poor correlations were observed between the Ross-Minton fit derived intrinsic viscosity versus the average intrinsic viscosity ([η]_avg_) with mid-concentration (a) and high concentration (b) viscosity profiles.** Error bars represent standard error [η]_avg_ values.


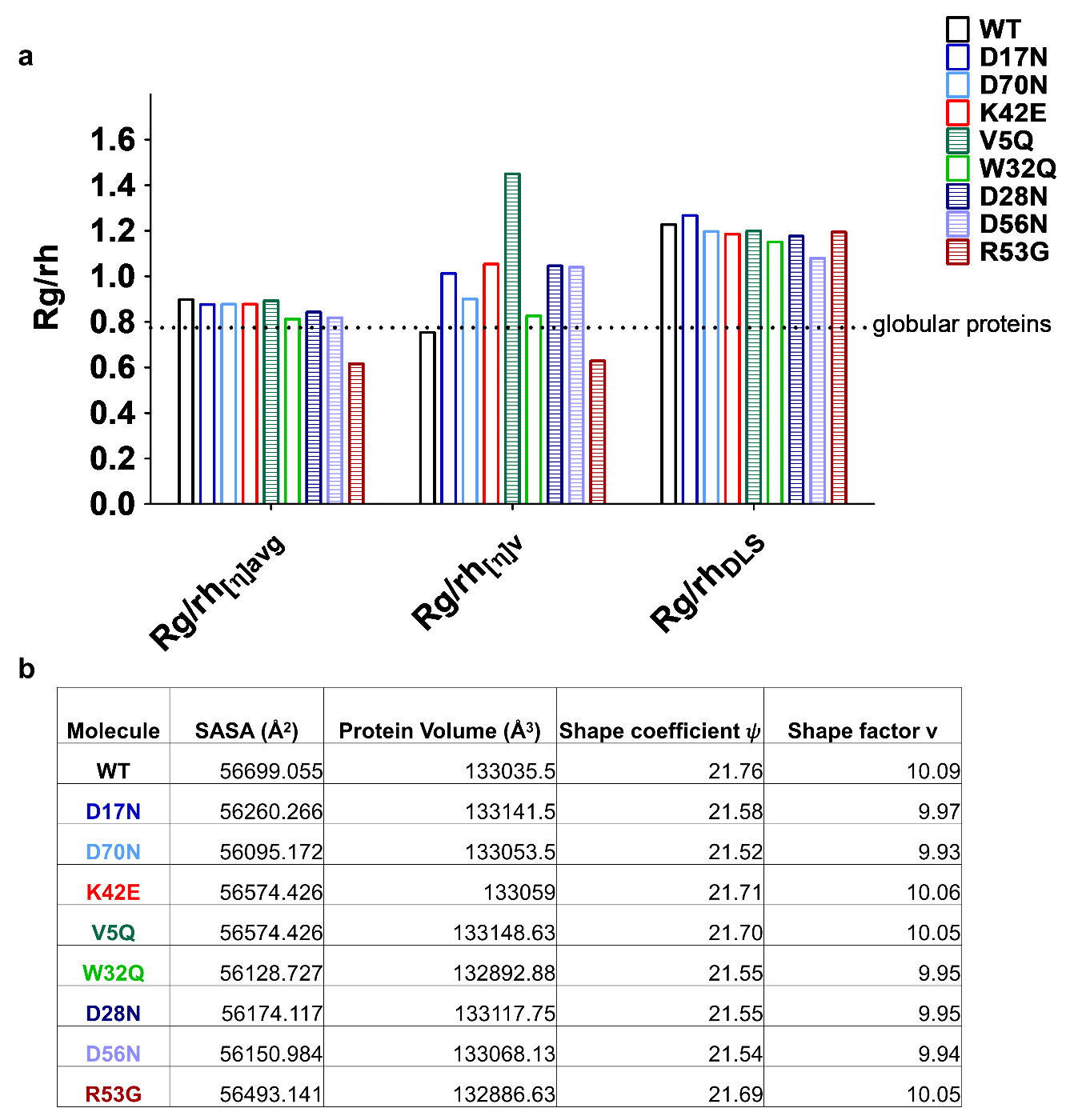


**Figure S7** **Shape factors of the anti-IL8 mAb panel.** Radius of gyration (Rg) was obtained from the HYDROPRO program (in shell mode) and three hydrodynamic radii (rh) were used as denominators; derived either from the linear average intrinsic viscosity [n]_avg_, the polynomial intrinsic viscosity [n]_v_ or DLS (**a**). A dotted line at 0.775 represents the expected ratio value for a spherical globular protein.^1^ Alternatively, computational solvent accessible surface area (SASA) and protein volumes were calculated from IgG homology constructs, and **Equation 12A and B** were used to compute shape coefficients and factors (v) (**b**).

**Table S5 Cross-correlation matrix for hydrodynamic parameters and high concentration viscosity (η) for anti-IL-8 mAb panel.** Pearson correlation coefficients (R) values are reported, and colour graded with positive correlations in blue and negative correlations in red. Correlations where R>0.8 are in white font. Abbreviations: [η]= intrinsic viscosity; [η]_H_ = [η] derived from linear η_red_/c Huggins plot; [η]_K_ = [η] derived from linear (Ln(η_rel_)/c)/c Kraemer plot; [η]_v_ = [η] derived from polynomial plot η_rel_/c plot; [η]_avg_ = average of [η]_H_ and [η]_K_; k_H_ = Huggins coefficient; k_K_ = Kraemer coefficient; k_exp_ = exponential constant from fitting η_rel_; rh = hydrodynamic radius derived from either intrinsic viscosity or from dynamic light scattering (DLS); Vh = hydrodynamic volume; ϕ = effective volume fraction derived from either intrinsic viscosity or DLS rh values; Z-ave = average measured hydrodynamic diameter from DLS; k_D_ = diffusion interaction parameter from DLS; B_22_ = second osmotic virial coefficient from DLS, ζ = mean zeta potential from electrophoretic light scattering; SASA = computed solvent-accessible surface area; Ψ = shape coefficient derived from SASA and computed protein volume; v = shape factor derived from Ψ.

**Predicting viscosity from molecular descriptors**

**Table S6 Ranking of anti-IL-8 molecules, in increasing viscosity order, according to Li, Sharma and Tomar predictions against experimental viscosity fitted across four models at 150 and 180 mg/mL.**

|  | **150 mg/mL** | | | | | **180 mg/mL** | | | | | | |
| --- | --- | --- | --- | --- | --- | --- | --- | --- | --- | --- | --- | --- |
|  | **Li score (cP)** | **Exponential growth fit viscosity (cP)** | **3-parameter exponential fit viscosity (cP)** | **Modified Ross Minton fit viscosity (cP)** | **Tomar fit viscosity (cP)** | **Sharma score (cP)** | **Tomar score (cP)** | **Exponential growth fit viscosity (cP)** | **3-parameter exponential fit viscosity (cP)** | **Modified Ross Minton fit viscosity (cP)** | **Tomar fit viscosity (cP)** | **Average knee (cP)** |
| *🡨 Increasing predicted viscosity* | **D17N** | **R53G** | **WT** | **WT** | **WT** | **D56N** | **D56N** | **R53G** | **WT** | **WT** | **WT** | **WT** |
|  | **D70N** | **K42E** | **D70N** | **R53G** | **D70N** | **D70N** | **D70N** | **W32Q** | **D70N** | **W32Q** | **D70N** | **D70N** |
|  | **D56N** | **W32Q** | **D17N** | **K42E** | **D17N** | **D17N** | **D17N** | **D70N** | **D17N** | **D28N** | **V5Q** | **V5Q** |
|  | **D28N** | **V5Q** | **V5Q** | **V5Q** | **V5Q** | **D28N** | **D28N** | **V5Q** | **V5Q** | **D70N** | **D17N** | **W32Q** |
|  | **W32Q** | **D70N** | **W32Q** | **W32Q** | **W32Q** | **V5Q** | **W32Q** | **WT** | **W32Q** | **R53G** | **W32Q** | **D17N** |
|  | **WT** | **WT** | **D28N** | **D28N** | **D28N** | **W32Q** | **V5Q** | **D17N** | **D28N** | **V5Q** | **D28N** | **D28N** |
|  | **V5Q** | **D28N** | **K42E** | **D70N** | **K42E** | **WT** | **WT** | **D28N** | **K42E** | **D17N** | **K42E** | **K42E** |
|  | **R53G** | **D17N** | **D56N** | **D17N** | **D56N** | **R53G** | **R53G** | **D56N** | **D56N** | **D56N** | **R53G** | **R53G** |
|  | **K42E** | **D56N** | **R53G** | **D56N** | **R53G** | **K42E** | **K42E** | **K42E** | **R53G** | **K42E** | **D56N** | **D56N** |

**Table S7** **Viscosity values from Ross-Minton fitting at 120 , 150 and 180 mg/mL with high-concentration and ultra-high-concentration data.** Viscosity values >30cP are shaded in red and <30cP are shaded in green. All molecules at 180 mg/mL had over the 30cP threshold when interpolating from ultra-high concentration regime data.

| **Concentration regime used:** | **High (<120 mg/mL)** | **Ultra-high (>120 mg/mL)** | **High (<120 mg/mL)** | **Ultra-high (>120 mg/mL)** | **High (<120 mg/mL)** | **Ultra-high (>120 mg/mL)** |
| --- | --- | --- | --- | --- | --- | --- |
| **Molecule** | **Ross-Minton fitted viscosity at 120 mg/mL (cP)** | | **Ross-Minton fitted viscosity at 150 mg/mL (cP)** | | **Ross-Minton fitted viscosity at 180 mg/mL (cP)** | |
| **WT** | 13.78 | 11.27 | 39.21 | 22.51 | 154.59 | 47.63 |
| **D17N** | 1302.84 | 27.61 | Too high | 52.27 | Too high | 94.22 |
| **D70N** | 7.28 | 19.68 | 11.92 | 39.31 | 19.76 | 77.88 |
| **K42E** | 166.68 | 4.59 | Too high | 25.30 | Too high | Too high |
| **V5Q** | 65.14 | 12.22 | 1584.29 | 28.75 | Too high | 79.76 |
| **W32Q** | 8.78 | 13.28 | 14.67 | 29.13 | 24.49 | 70.02 |
| **D28N** | 225.76 | 14.10 | Too high | 31.34 | Too high | 76.21 |
| **D56N** | 17.32 | 101.84 | 77.56 | 233.25 | 892.44 | 491.72 |
| **R53G** | >20,000 | 9.21 | Too high | 23.03 | Too high | 79.43 |

**Machine Learning Tools for Viscosity Prediction**

Lai et al. used classification models of 27 approved mAbs to generate a decision tree for categorising viscosity risk at 150 mg/mL based on computed net charge and the number of hydrophilic and hydrophobic residues in the Fv.^2^ We found all our molecules were classed at high viscosity risks (≥30 cP) using the same thresholds. This agrees with experimental viscosities of the anti-IL-8 mAb panel, which were >30cP with the three-parameter exponential and Tomar model fits at 150 mg/mL, using the high concentration data (100% true negatives using either a 20 cP or 30 cP cut-off, **Supplementary Table S7**).

**Table S8 Confusion matrix demonstrating number of molecules with predicted low or high viscosity from use of ensemble charge (ens_charge)**. Using the high concentration regime data, three out of the nine molecules would be classified as low viscosity despite >30cP experimental viscosities at 120 mg/mL. Using the ultra-high concentration regime data, two molecules would be classified as low viscosity despite >30cP experimental viscosity reported/ at 120 mg/mL.

| **High (<120 mg/mL) concentration regime** | | |
| --- | --- | --- |
| *No. molecules* | **Low viscosity (<30 cP) at 120 mg/mL** | **High viscosity (>30 cP) at 120 mg/mL** |
| **Predicted low viscosity (ens_charge >+2)** | 4 | 3 |
| **Predicted high viscosity (ens_charge <+2)** | 0 | 2 |
| **Ultra-high (>120 mg/mL)** **concentration regime** | | |
| *No. molecules* | **Low viscosity (<30 cP) at 120 mg/mL** | **High viscosity (>30 cP) at 120 mg/mL** |
| **Predicted low viscosity (ens_charge >+2)** | 5 | 2 |
| **Predicted high viscosity (ens_charge <+2)** | 2 | 0 |

**Figure S 8** All anti-IL-8 mAb molecules were classified to have high viscosity at 150 mg/mL Net charge of full IgG homology constructs and hydrophobic (A, F, I, L, M, P, V, W) and hydrophilic (S, T, N, Q, Y, K, R, H, D, E) residue counts in the Fv were obtained to generate the High Viscosity Index (HVI) with the equation $\frac{N_{hyph-N_{hyd}}}{N_{res}}\times100$ Thresholds from original model development for low viscosity (<30cP at 150 mg/mL) were <34 C net charge and <17.3 HVI if the net charge >12 C.

Makowski *et al.* developed an alternative decision tree for viscosity classification using three molecular properties from 79 proprietary mAbs.^3^ Structural-based isoelectric point (*pI_3D*), largest hydrophobic patch and number of negative patches from Fv homology models were strongly correlated features used for the classifier algorithm. In our study, we saw that only negative-patch targeting mutants (DàN) reached the pI threshold of ≥6.3, classifying these molecules with low viscosity predictions (<20cP at >100 mg/mL) (**Figure 9**). Although all molecules had ‘acceptable’ values for the largest hydrophobic area and number of negative patches, WT, hydrophobic patch targeting and positive patch targeting molecules were classed to have high viscosity due to lower pI values. This classification had lower prediction accuracy to experimental compared to the Lai *et al.* triaging tool, with 56% true negatives and 44% false positives for the three-parameter exponential and Tomar fitted viscosities (**Supplementary Table S8**).

**Table S9 Confusion matrices of viscosity classification of anti-IL-8 molecules from Lai et al. decision tree**^2^ **versus experimental viscosity data.** The experimental data used are the interpolated viscosities at 120 mg/mL and 150 mg/mL from all fitting models used (modified Ross-Minton, growth exponential, 3- parameter exponential and Tomar models). Two cut-off points were used (20 or 30 cP) as the ‘low viscosity’ threshold. Top left quadrant of each matrix represents true positives, bottom left quadrant represents false negatives, top right quadrant represents false positives and bottom right quadrant represents true negatives. True positive or negative values are in green font and false positive or negatives are in red font.

|  |  | **Viscosity classification at 120 mg/mL** | | | |  | **Viscosity classification at 150 mg/mL** | | | |
| --- | --- | --- | --- | --- | --- | --- | --- | --- | --- | --- |
|  |  | **20cP cut off** | | **30 cP cut off** | |  | **20cP cut off** | | **30 cP cut off** | |
|  |  | **Modified Ross-Minton fitted viscosities** | | | | | | | | |
|  | *No. molecules* | **Low viscosity (<20 cP) at 120 mg/mL** | **High viscosity (>20 cP) at 120 mg/mL** | **Low viscosity (<30 cP) at 120 mg/mL** | **High viscosity (>30 cP) at 120 mg/mL** | *No. molecules* | **Low viscosity (<20 cP) at 150 mg/mL** | **High viscosity (>20 cP) at 150 mg/mL** | **Low viscosity (<30 cP) at 150 mg/mL** | **High viscosity (>30 cP) at 150 mg/mL** |
|  | **Predicted low viscosity (Lai et al.)** | **0** | **0** | **0** | **0** | **Predicted low viscosity (Lai et al.)** | **0** | **0** | **0** | **0** |
|  | **Predicted high viscosity (Lai et al.)** | **7** | **2** | **8** | **1** | **Predicted high viscosity (Lai et al.)** | **0** | **9** | **5** | **4** |
|  |  | **Growth exponential fitted viscosities** | | | | | | | | |
|  | **Predicted low viscosity (Lai et al.)** | **0** | **0** | **0** | **0** | **Predicted low viscosity (Lai et al.)** | **0** | **0** | **0** | **0** |
|  | **Predicted high viscosity (Lai et al.)** | **6** | **3** | **7** | **2** | **Predicted high viscosity (Lai et al.)** | **2** | **7** | **4** | **5** |
|  |  | **3-parameter exponential fitted viscosities** | | | | | | | | |
|  | **Predicted low viscosity (Lai et al.)** | **0** | **0** | **0** | **0** | **Predicted low viscosity (Lai et al.)** | **0** | **0** | **0** | **0** |
|  | **Predicted high viscosity (Lai et al.)** | **4** | **5** | **7** | **2** | **Predicted high viscosity (Lai et al.)** | **0** | **9** | **0** | **9** |
|  |  | **Tomar fitted viscosities** | | | | | | | | |
|  | **Predicted low viscosity (Lai et al.)** | **0** | **0** | **0** | **0** | **Predicted low viscosity (Lai et al.)** | **0** | **0** | **0** | **0** |
|  | **Predicted high viscosity (Lai et al.)** | **1** | **8** | **6** | **3** | **Predicted high viscosity (Lai et al.)** | **0** | **9** | **0** | **9** |

**Figure S9** **Negative patch targeting mutants predicted to have lower viscosity using the Makowski decision tree.**^3^ Fv homology constructs were used to derive isoelectric points (pI_3D), largest hydrophobic patch area (Å^2^) and number of negative patches. Cut-offs from original model development were pI_3D ≥6.3, largest hyd patch <261 Å^2^ and N_neg patches >5.8. Only D🡪N mutants passed the pI criteria.

**Table S10 Confusion matrix of viscosity classification of anti-IL-8 molecules from Makowski et al. decision tree**^3^ **versus experimental viscosity data.** The experimental data used are the interpolated viscosities at 120 mg/mL and 150 mg/mL from all fitting models used (modified Ross-Minton, growth exponential, 3- parameter exponential and Tomar models). Two cut-off points were used (20 or 30 cP) as the ‘low viscosity’ threshold. Top left quadrant of each matrix represents true positives, bottom left quadrant represents false negatives, top right quadrant represents false positives and bottom right quadrant represents true negatives. True positive or negative values are in green font and false positive or negatives are in red font.

|  | **Viscosity classification at 120 mg/mL** | | | |  | **Viscosity classification at 150 mg/mL** | | | |
| --- | --- | --- | --- | --- | --- | --- | --- | --- | --- |
|  | **20cP cut off** | | **30 cP cut off** | |  | **20cP cut off** | | **30 cP cut off** | |
|  | **Modified Ross-Minton fitted viscosities** | | | | | | | | |
| *No. molecules* | **Low viscosity (<20 cP) at 120 mg/mL** | **High viscosity (>20 cP) at 120 mg/mL** | **Low viscosity (<30 cP) at 120 mg/mL** | **High viscosity (>30 cP) at 120 mg/mL** | *No. molecules* | **Low viscosity (<20 cP) at 150 mg/mL** | **High viscosity (>20 cP) at 150 mg/mL** | **Low viscosity (<30 cP) at 150 mg/mL** | **High viscosity (>30 cP) at 150 mg/mL** |
| **Predicted low viscosity (Makowski et al.)** | **2** | **2** | **3** | **1** | **Predicted low viscosity (Makowski et al.)** | **0** | **4** | **0** | **4** |
| **Predicted high viscosity (Makowski et al.)** | **5** | **0** | **5** | **0** | **Predicted high viscosity (Makowski et al.)** | **0** | **5** | **5** | **0** |
|  | **Growth exponential fitted viscosities** | | | | | | | | |
| **Predicted low viscosity (Makowski et al.)** | **2** | **2** | **2** | **2** | **Predicted low viscosity (Makowski et al.)** | **0** | **4** | **0** | **4** |
| **Predicted high viscosity (Makowski et al.)** | **5** | **1** | **5** | **0** | **Predicted high viscosity (Makowski et al.)** | **0** | **5** | **1** | **1** |
|  | **3-parameter exponential fitted viscosities** | | | | | | | | |
| **Predicted low viscosity (Makowski et al.)** | **3** | **1** | **3** | **1** | **Predicted low viscosity (Makowski et al.)** | **0** | **4** | **0** | **4** |
| **Predicted high viscosity (Makowski et al.)** | **2** | **3** | **3** | **1** | **Predicted high viscosity (Makowski et al.)** | **0** | **5** | **0** | **5** |
|  | **Tomar fitted viscosities** | | | | | | | | |
| **Predicted low viscosity (Makowski et al.)** | **0** | **4** | **3** | **1** | **Predicted low viscosity (Makowski et al.)** | **0** | **4** | **0** | **4** |
| **Predicted high viscosity (Makowski et al.)** | **1** | **4** | **3** | **2** | **Predicted high viscosity (Makowski et al.)** | **0** | **5** | **0** | **5** |


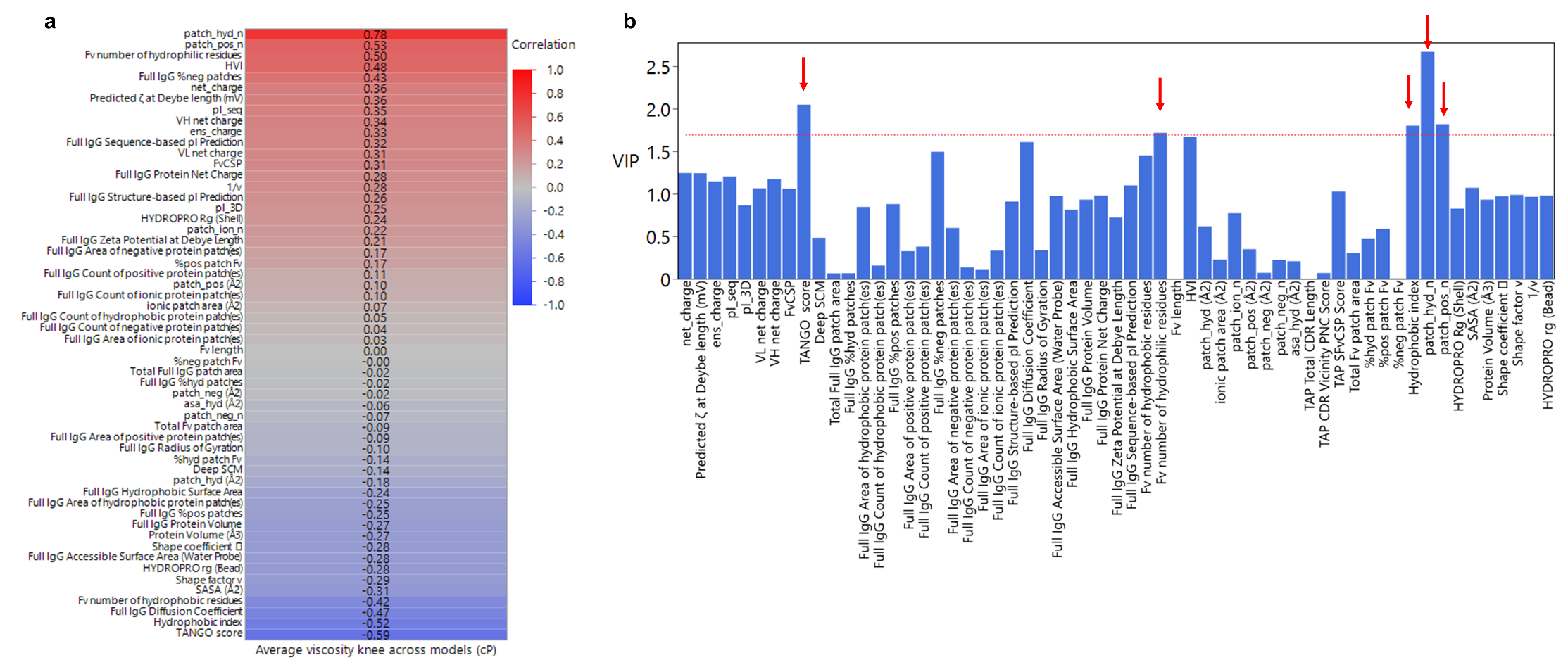


**Figure S10 Correlations (a) and variable importance plot (VIP) (b) of averaged knee of viscosity profiles for anti-IL-8 panel to computational molecular descriptors.** Highest correlated descriptors (R≥0.5) which are marked with red arrows on the VIP were used in partial least squares regression for predictive modelling. Partial least squared modelling was used to generate the VIP and a threshold of 1.7 (red dotted line) was set.

**Table S11 Molecular descriptors from four in-house proprietary mAbs were used to test regression model developed from anti-IL8 panel.**

| **Molecule** | **Hydrophobic index** | **TANGO** | **Fv number of hydrophilic residues** | **Fv number of hydrophobic residues** | **HVI** | **Full IgG %neg patches** | **Full IgG diffusion coefficient** | **Count of hydrophobic protein patch(es)** | **Count of positive protein patch(es)** |
| --- | --- | --- | --- | --- | --- | --- | --- | --- | --- |
| **Ab1** | 1.048 | 683.77 | 118 | 84 | 14.72 | 21.07% | 4.70E-07 | 11 | 11 |
| **Ab2** | 1.087 | 863.13 | 120 | 81 | 16.81 | 27.55% | 4.70E-07 | 10 | 7 |
| **Ab3** | 1.124 | 384.17 | 125 | 85 | 16.81 | 22.88% | 4.70E-07 | 9 | 10 |
| **Ab4** | 1.069 | 770.32 | 124 | 80 | 18.88 | 23.82% | 4.80E-07 | 11 | 11 |

**References**

(1) Abdelmohsen, L. K. E. A.; Rikken, R. S. M.; Christianen, P. C. M.; Van Hest, J. C. M.; Wilson, D. A. Shape Characterization of Polymersome Morphologies via Light Scattering Techniques. *Polymer* **2016**, *107*, 445–449. https://doi.org/10.1016/j.polymer.2016.06.067.

(2) Lai, P.-K.; Fernando, A.; Cloutier, T. K.; Gokarn, Y.; Zhang, J.; Schwenger, W.; Chari, R.; Calero-Rubio, C.; Trout, B. L. Machine Learning Applied to Determine the Molecular Descriptors Responsible for the Viscosity Behavior of Concentrated Therapeutic Antibodies. *Molecular Pharmaceutics* **2021**. https://doi.org/10.1021/acs.molpharmaceut.0c01073.

(3) Makowski, E. K.; Chen, H.-T.; Wang, T.; Wu, L.; Huang, J.; Mock, M.; Underhill, P.; Pelegri-O’Day, E.; Maglalang, E.; Winters, D.; Tessier, P. M. Reduction of Monoclonal Antibody Viscosity Using Interpretable Machine Learning. *mAbs* **2024**, *16* (1), 2303781. https://doi.org/10.1080/19420862.2024.2303781.
